# Imidazolium-Based Sulfonating Agent to Control the Degree of Sulfonation of Aromatic Polymers and Enable Plastics-to-Electronics Upgrading

**DOI:** 10.1021/jacsau.4c00355

**Published:** 2024-07-03

**Authors:** Chun-Yuan Lo, Kelsey P. Koutsoukos, Dan My Nguyen, Yuhang Wu, David Alejandro Angel Trujillo, Tabitha Miller, Tulaja Shrestha, Ethan Mackey, Vidhika S. Damani, Uddhav Kanbur, Robert Opila, David C. Martin, David Kaphan, Laure V. Kayser

**Affiliations:** † Department of Chemistry and Biochemistry, University of Delaware, Newark, Delaware 19716, United States; ‡ Department of Materials Science and Engineering, University of Delaware, Newark, Delaware 19716, United States; § Chemical Sciences and Engineering Division, Argonne National Laboratories, Lemont, Illinois 60439, United States; ∥ Department of Biomedical Engineering, University of Delaware, Newark, Delaware 19716, United States

**Keywords:** sulfonation, plastic upgrading, polyelectrolytes, electrophilic aromatic substitution, PEDOT:PSS, organic electrochemical transistors, hybrid solar cells

## Abstract

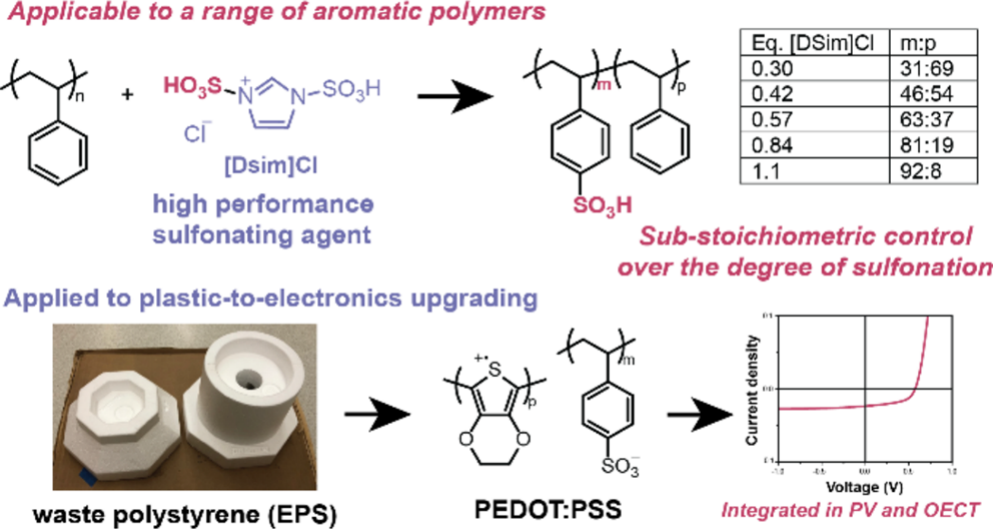

The accumulation of plastic waste in the environment is a growing environmental, economic, and societal challenge. Plastic upgrading, the conversion of low-value polymers to high-value materials, could address this challenge. Among upgrading strategies, the sulfonation of aromatic polymers is a powerful approach to access high-value materials for a range of applications, such as ion-exchange resins and membranes, electronic materials, and pharmaceuticals. While many sulfonation methods have been reported, achieving high degrees of sulfonation while minimizing side reactions that lead to defects in the polymer chains remains challenging. Additionally, sulfonating agents are most often used in large excess, which prevents precise control over the degree of sulfonation of aromatic polymers and their functionality. Herein, we address these challenges using 1,3-disulfonic acid imidazolium chloride ([Dsim]Cl), a sulfonic acid-based ionic liquid, to sulfonate aromatic polymers and upgrade plastic waste to electronic materials. We show that stoichiometric [Dsim]Cl can effectively sulfonate model polystyrene up to 92% in high yields, with minimal defects and high regioselectivity for the *para* position. Owing to its high reactivity, the use of substoichiometric [Dsim]Cl uniquely allows for precise control over the degree of sulfonation of polystyrene. This approach is also applicable to a wide range of aromatic polymers, including waste plastic. To prove the utility of our approach, samples of poly(styrene sulfonate) (PSS), obtained from either partially sulfonated polystyrene or expanded polystyrene waste, are used as scaffolds for poly(3,4-ethylenedioxythiophene) (PEDOT) to form the ubiquitous conductive material PEDOT:PSS. PEDOT:PSS from plastic waste is subsequently integrated into organic electrochemical transistors (OECTs) or as a hole transport layer (HTL) in a hybrid solar cell and shows the same performance as commercial PEDOT:PSS. This imidazolium-mediated approach to precisely sulfonating aromatic polymers provides a pathway toward upgrading postconsumer plastic waste to high-value electronic materials.

## Introduction

Aromatic polymers, such as polystyrene (PS), styrene–ethylene–butadiene–styrene (SEBS), polyether sulfone (PES), and poly(ethylene terephthalate) (PET) are widely used in daily life in packaging, biomedical devices, synthetic rubbers, and automotive parts. However, the continuous growth in the production of these aromatic plastics leads to increased amounts of waste being disposed of in landfills or the environment. As such, plastic waste represents a mounting environmental, economic, and societal challenge.^1^ Current plastic waste mitigation approaches include mechanical recycling or chemical recycling by conversion of polymeric materials into monomers, fuels, or chemical precursors.^2−4^ Plastic recycling strategies, however, are often not economically viable due to low plastic manufacturing costs outcompeting the value of recycled plastics or small molecule products.^5^ Instead, creating value-added materials from aromatic plastic waste (i.e., plastic upcycling or upgrading) could be a more effective approach. As such, an increasing number of studies have focused on the functionalization of plastics.^6−11^ Owing to the innate reactivity of its aromatic phenyl ring, PS has often been used as a model material for further functionalization.^8,12,13^ PS can be functionalized by chlorosulfonation of PS resin with a degree of substitution between 50 and 66%,^14^ Friedel–Crafts acylation to generate a PS copolymer with 10% of units possessing an acyl chloride,^12^ or the use of perfluoroacyl peroxides as precursors to perfluoroalkyl radicals.^15^ While efficient, each of these functionalization methods requires harsh reagents and conditions, which can cause significant polymer degradation.^8,13^ Hence, milder methods that afford high degrees of functionalization without degradation or defects for the conversion of PS to higher-value products are needed.

**Figure 1:**
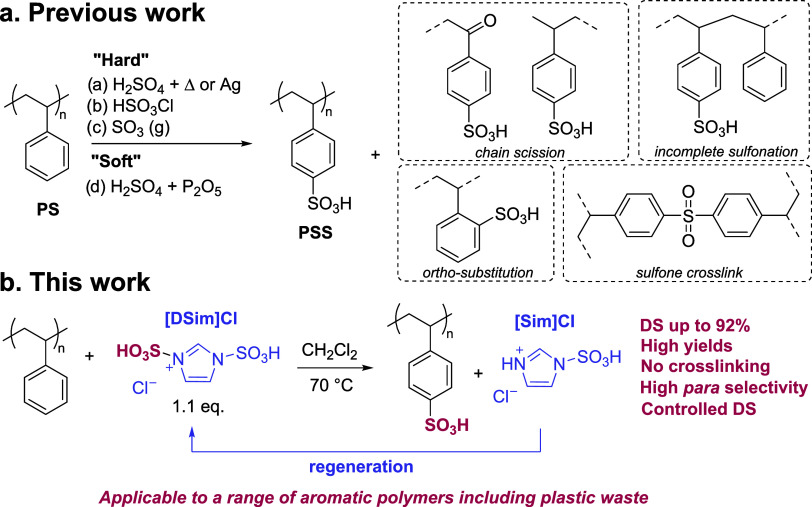
Sulfonation of polystyrene (PS) to poly(styrene sulfonate) (PSS). (a) Previous strategies showed possible defects resulting from side reactions. (b) This work used a stoichiometric and regenerable imidazolium salt, [Dsim]Cl.

Polystyrene sulfonate (PSS) is a high-value material used in many applications, such as ion-exchange resins (e.g., for metal separation, water softening, and water decontamination processes),^16^ membranes (e.g., extraction of lithium ions from salt-lake brine),^17^ pharmaceuticals (e.g., high potassium in blood treatment),^18^ and electronics (e.g., antistatic coatings and conducting polymers).^19^ Several approaches are available for transforming PS into PSS, which are broadly classified as either “hard” or “soft” sulfonation techniques (Figure 1a).^20,21^ “Hard” refers to the use of strong sulfonation agents/conditions (fuming sulfuric acid^22^ and chlorosulfonic acid^23^), which often lead to high degrees of sulfonation (DS) over 90% but come at the expense of side reactions such as oxidation, chain scission, or the formation of sulfone crosslinks. “Soft” sulfonation, such as Vink’s method, uses sulfuric acid with phosphorus pentoxide and cyclohexane as a solvent to limit defects.^24^ Coughlin et al. showed that using concentrated sulfuric acid and carefully controlling the temperature, the benefits of the “hard” and “soft” sulfonation methods can be combined to obtain DS > 96% without side reactions.^21^ Drawbacks, however, persist. First, the particle size of PS must be small (<100 μm), precluding the direct sulfonation of commodity PS, particularly its waste products, which are often crosslinked and would need to be mechanically broken down. Second, the reaction is not 100% regioselective for the *para* position as 6% −SO_3_ was found in the *meta* position. Lastly, a common problem with all of these sulfonation methods is the difficulty in controlling DS, which is an essential parameter to tune the properties of sulfonated polymers for varying applications.^21^ Past approaches to control DS have been limited to manipulating the temperature and reaction time or using a sulfonating agent (e.g., acetyl sulfate) in a solvent. For the former, DS is controlled by reaction kinetics and therefore needs adjusting for each polymer of interest, and higher temperatures also lead to more degradation and defects in the sulfonated polymer.^21,25,26^ When using acetyl sulfate, a major problem is that DS is always lower than the predicted value because the sulfonating agent is not sufficiently active under dilute conditions.^27,28^ To the best of our knowledge, no method has been reported to precisely control DS using stoichiometric sulfonating agents.

In this article, we addressed these limitations to obtain PSS and other sulfonated aromatic polymers in high yields, DS, purity, and regioselectivity for the *para* position using an ionic liquid as a sulfonating agent (Figure 1b), combining the advantages of both “hard” and “soft” sulfonation methods. Importantly, owing to the high reactivity of the sulfonating agent, we were able to precisely control DS.

Ionic liquids have received considerable attention as reagents or catalysts in the context of eco-friendly synthesis because of their negligible vapor pressure, low volatility, nonflammability, high thermal stability, and ability to dissolve a wide range of industrial materials. Of particular interest, Moosavi-Zare et al. previously showed that toluene can be sulfonated by 1,3-disulfonic acid imidazolium chloride ([Dsim]Cl), a sulfonic acid-based ionic liquid, with >99% regioselectivity for the *para* position, through an electrophilic aromatic substitution.^29^ However, functionalizing polymers, as opposed to small molecules, presents unique challenges, including maintaining polymer chain integrity and controlling the degree of functionalization while avoiding side reactions that may lead to defects in the polymer chain (e.g., incomplete functionalization, undesired functional groups, or crosslinking) or chain scission. In the specific case of the sulfonation of polymers, these challenges have not been fully addressed because of either too harsh or too mild sulfonating agents used. Notably, the need for excess sulfonating agents often leads to sulfone crosslinks (Figure 1a).^23^ Herein, we demonstrate the use of [Dsim]Cl as a mild, yet effective, sulfonating agent for PS. The key optimization strategies to translate this sulfonation with [DSim]Cl to polymers were to use a good solvent for the polymer to be sulfonated and maintain mild reaction temperatures and a 1:1 molar ratio of [Dsim]Cl to styrene. Using optimized conditions, PSS and other sulfonated polymers can be prepared from aromatic polymers with controllable DS and selectivity for the *para* position, and without chain scission or crosslinking. We found that the by-product of the reaction, (1-sulfonic acid imidazolium) chloride ([Sim]Cl), can be readily recovered in the form of protonated imidazolium to regenerate [Dsim]Cl. Given the high reactivity of the sulfonating agent, using substoichiometric amounts also allowed for control over DS without changing the temperature or reaction time. To demonstrate the utility of our approach and its applicability, we prepared P(SS-*co*-S) with varying DS to use as a matrix for the synthesis of poly(3,4-ethylenedioxythiophene) to form PEDOT:P(SS-*co*-S), a conductive polyelectrolyte complex. We found that DS was strongly correlated to electronic conductivity. Most importantly, we sulfonated expanded PS (EPS) from waste packaging. PSS thereby obtained (DS = 92%) was used to synthesize PEDOT:PSS. We found that PEDOT:PSS derived from plastic waste performed similarly to commercially available samples in silicon-based hybrid solar cells and organic electrochemical transistors (OECTs). This new sulfonation strategy opens avenues toward high-quality sulfonated polymers, including from postconsumer waste plastics, that can be used as materials for electronics, i.e., plastics-to-electronics upgrading.

## Results and Discussion

**Figure 2:**
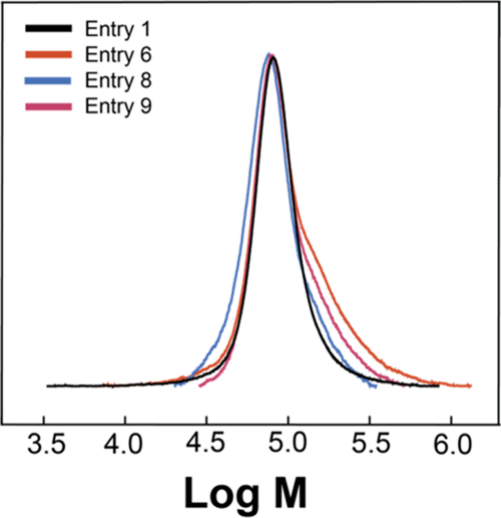
Molecular weight distribution (log *M*) obtained from SEC of selected PSS samples obtained by sulfonation of PS from Table 1, Entries 1 (Vink sulfonation), 6, 8, and 9 ([Dsim]Cl sulfonation).

The sulfonating agent used for this study, [Dsim]Cl, was synthesized according to previously reported procedures by reacting imidazole with chlorosulfonic acid (details in Supporting Information (SI) page S3).^29^ Under optimized conditions, we limited the amount of monosulfonated imidazolium, (1-sulfonic acid imidazolium) chloride ([Sim]Cl), side product to ∼10 mol % (Figure 1b). This impurity was not problematic as it did not participate in the reaction but was accounted for when calculating the molar ratio of [Dsim]Cl added to the reaction. To optimize the reaction conditions for the sulfonation of aromatic polymers with [Dsim]Cl, we started by evaluating DS of a commercially available PS (*M*_*n*_ = 35 kg mol^–1^, *Đ* = 1.18). A PS with a narrow molecular weight distribution was selected to monitor for crosslinking or chain cleavage defects. As a benchmark, we first sulfonated this polymer using the Vink sulfonation method,^24^ a “soft” sulfonation method involving excess sulfuric acid with phosphorus pentoxide in cyclohexane (Table 1, **Entry 1**). While we obtained a DS of 91%, the yield was only 64% likely due to losses during the aqueous workup, where PS with high DS was more efficiently extracted in water than PS with a lower DS (less water soluble). Then, we started our study with [Dsim]Cl by establishing which solvent would be best suited by performing the reaction at 50 °C for 4 h with 1.4 equiv of [Dsim]Cl per styrene (S) repeat unit ([Dsim]Cl/S). As [Dsim]Cl is a liquid above room temperature, we attempted the sulfonation without additional solvent (Table 1, **Entry 2**). Under these neat conditions, however, no sulfonation was recorded, likely due to the insolubility of PS in [Dsim]Cl. Next, we explored nonpolar and polar solvents that are commonly used to dissolve PS. When tetrahydrofuran (THF) (Table 1, **Entry 3**) and cyclohexane (Table 1, **Entry 4**), were used as solvents, DS of <1 and 71%, and yields of <1 and 40%, respectively, were obtained. These low DS and yields are likely due to the poor solubility of the [Dsim]Cl sulfonating agent.^30^ When switching to chlorinated solvents, such as chloroform (CHCl_3_) (Table 1, **Entry 5**) and dichloromethane (DCM) (Table 1, **Entry 6**), we obtained higher yields and DS, with the best results in DCM with a 65% yield and an 86% DS. With the ideal solvent in hand, we then investigated the effect of temperature. When performing the reaction at room temperature (Table 1, **Entry 7**), we found that the yield slightly increased (84%) and DS slightly decreased (79%). Importantly, we found that under these last two conditions, *Đ* reached 1.5 with a high molecular weight shoulder visible in the size exclusion chromatography (SEC) chromatogram when compared with PSS obtained from the soft Vink sulfonation (Figure 2). This shoulder can be attributed to interpolymer chain crosslinking defects, most likely sulfone crosslinks (Figure 1a), which are common in sulfonation reactions at high sulfonating agent molar ratios.^23^ As this defect was present only in low concentration, we observed a small fraction of PSS with roughly double the molecular weight (two polymer chains crosslinking resulting in high molecular weight shoulder) but not the formation of an insoluble network. This crosslinking problem was addressed by decreasing the molar ratio of [Dsim]Cl/S from 1.4 to 1.1 (Table 1, **Entry 8**). While a slightly lower yield and DS were obtained at 50 °C, the dispersity dropped back down to 1.24 (Figure 2). By increasing the reaction temperature to 70 °C and maintaining a ∼1:1 [Dsim]Cl/S ratio (Table 1, **Entry 9**), DS increased to 92% and yielded 87%, while keeping a dispersity of 1.24. Increasing the concentration, however, led to a lower DS (73%), likely due to premature precipitation (Table 1, **Entry 10**). This ∼1:1 ratio of sulfonating agent:S was much lower than previously reported methods that required a large excess of sulfonating agent (e.g., Vink sulfonation, Table 1, **Entry 1**),^24^ yet resulted in similar DS and higher yields. This increase in yield can be attributed to the workup approach, which in our case did not require large amounts of ice to quench excess sulfuric acid. When compared with previous established procedures in the literature (Table S1), such as sulfonation with sulfur trioxide with P_2_O_5_,^31,32^ acetyl sulfate,^33^ or chlorosulfonic acid,^34^ the use of [Dsim]Cl resulted in higher yields, DS, or both, without compromising on the structure of PSS. Additionally, this approach worked well on higher MW PS with *M*_*n*_ = 120 kg mol^–1^ and *Đ* = 1.13, following the same conditions as in **Entry 9**, affording a similar DS (92%) and maintaining a low dispersity (1.24) (Table 1, **Entry 11**). In most cases, we noticed a discrepancy between the measured and theoretical molecular weights of PSS. In the cases where PS was 35 kg mol^–1^, we measured lower values by SEC than the theoretical *M*_*n*_ value for PSS (69 kg mol^–1^). While with the higher molecular weight PS (120 kg mol^–1^), PSS measured value was lower than the theoretical (237 kg mol^–1^). These differences are not uncommon in the literature,^21^ but typically not discussed. We posit that these differences could arise from either experimental error in SEC owing to the calibration standards being fully sulfonated or loss of low or high molecular fractions during the workup of PSS. We also endeavored to evaluate the regiochemical outcomes of the sulfonation with [DSim]Cl. A previous report on sulfonation with pure sulfuric acid showed that the sulfonation favored *para* over *meta* substitution with roughly a 94:6 ratio.^21^ Using quantitative ^13^C NMR, we found that the sulfonation using [Dsim]Cl leads to 99.5 ± 0.3% selectivity for the para position (Figure S2). The Vink sulfonation was similarly regioselective at 98.9 ± 0.2%.

**Table 1:**
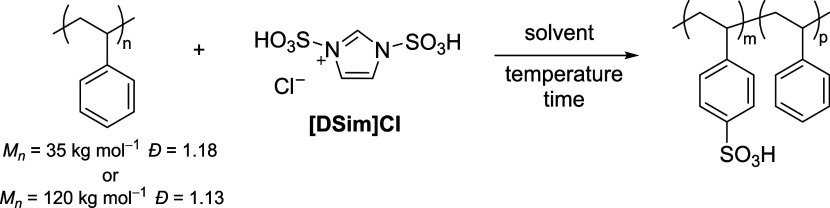
Optimization of the Yield and Degree of Sulfonation for the Sulfonation of PS Using [Dsim]Cl

entry	[PS]^a^ (g mL^–1^)	solvent	[Dsim]Cl/S^b^ molar ratio	time (h)	temp. (°C)	DS^c^ (%)	Isolated yield (%)	*M*_*n*_^d^ (kg mol^–1^)	*Đ*^d^
1^e^	0.005	cyclohexane	N.A.	5	50	91	64	74.8	1.22
2	1	neat	1.4	4	50	<1^f^	<1		
3	0.003	THF	1.4	4	50	<1^f^	<1		
4	0.003	cyclohexane	1.4	4	50	71	40		
5	0.003	CHCl_3_	1.4	4	50	64	64		
6	0.003	DCM	1.4	4	50	86	65	85.2	1.46
7	0.003	DCM	1.4	4	r.t.	79	84	89.2	1.53
8	0.003	DCM	1.1	4	50	80	64	76.5	1.24
9	0.003	DCM	1.1	4	70	92	87	86.3	1.24
10	0.017	DCM	1.1	4	70	73	65	83.9	1.18
11	0.003^g^	DCM	1.1	4	70	92	77	207	1.24

a All of the optimization experiments were done using PS with *M*_*n*_ = 35 kg mol^–1^ and *Đ* = 1.18, except in **Entry 11**.

b S = equivalent of styrene repeat unit.

c Determined by UV–vis titration using 0.1 M NaOH.^35,36^

d Obtained by SEC in a water buffer:methanol 80:20 calibrated against PSSNa standards using a refractive index detector.

e Vink’s approach, sulfonation in neat sulfuric acid.

f Determined by Fourier transform infrared spectroscopy (FTIR).

g Using higher *M*_*n*_ PS (*M*_*n*_ = 120 kg mol^–1^, *Đ* = 1.13).

**Figure 3:**
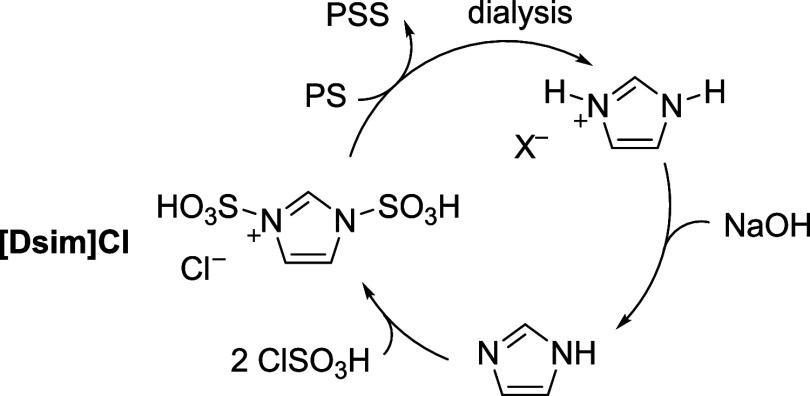
Overview of the process to recover and regenerate [Dsim]Cl.

From an industrial synthesis point of view, the use of imidazole-based reagents may be too costly compared with sulfuric acid, outweighing the benefits of the obtained high-purity sulfonated products. Additionally, while we have yet to perform a full life cycle analysis of the process, the use of nitrogen-based reagents often leads to higher CO_2_ emissions than sulfuric acid derivatives.^37,38^ To address these economic and environmental concerns, we investigated the recovery and regeneration of [Dsim]Cl (Figure 3). After the sulfonation of PS, PSS was extracted from the reaction mixture and purified by dialysis. During the dialysis (MWCO 3500), the water-soluble small molecule by-products of the reaction leach out of the dialysis tube. The major by-product after this workup appeared to be a symmetrical protonated imidazolium (Figure S3),^39^ which can be converted back quantitatively to imidazole (Figure S4) by the addition of NaOH and subsequently extracted in ethyl acetate. After evaporation of the solvent, pure imidazole is obtained, which can be reused for the synthesis of [Dsim]Cl. Using this approach, we were able to recover 68% of the imidazole. The remaining 32% was likely trapped in PSS and discarded with the acidic resin during subsequent PSS workup steps. This imidazole was reacted with chlorosulfonic acid to produce [Dsim]Cl in a yield of 90%, giving an overall recovery of 61%. We note that the recovered [Dsim]Cl contained 17% [Sim]Cl side product (Figure S5), which was not detrimental to the reaction—we have performed the sulfonation with up to 31% [Sim]Cl impurity without negative effect on the DS—but should be accounted for when calculating the stoichiometric ratio of [Dsim]Cl/S. Lastly, DCM could also potentially be recycled as it did not contain any residual reagents or by-products as observed by ^1^H NMR.

As mentioned in the introduction, controlling DS is an outstanding challenge in sulfonated polymers, highly dependent on the reaction time and temperature in the presence of an excess sulfonating agent. Having obtained high yields and DS of PS, we checked whether we could regulate DS by controlling the molar ratio of [Dsim]Cl/S. Using a temperature of 70 °C and a reaction time of 4 h, we used substoichiometric amounts of [Dsim]Cl for the sulfonation of PS ([PS] = 3 mg mL^–1^) (Table 2 and Figure S6). In all cases, DS of the isolated P(SS-*co*-S) products was within a 6% margin of error from the expected DS, demonstrating that [Dsim]Cl can control DS with relatively high accuracy, without otherwise changing the reaction conditions, and while maintaining the molecular weight and dispersity. This level of control over DS allowed us to probe its effect on the conductivity of PEDOT:P(SS-*co*-S) polyelectrolyte complexes. We synthesized these complexes by oxidative polymerization of 3,4-ethylene dioxythiophene (EDOT) in water in the presence of P(SS-*co*-S) matrices with varying DS of 31, 46, 63, 81, and 92% (details in SI, page S10). With the exception of the 31% DS sample, all of the resulting PEDOT:P(SS-*co*-S) complexes formed stable colloidal dispersions in water. Thin films of these complexes, neat or with dimethyl sulfoxide (DMSO) and dodecyl benzene sulfonic acid (DBSA) secondary dopants,^40−42^ were prepared by spin-coating and their conductivity was measured by a four-point probe. We found that for both the neat and the doped samples, the conductivity increased gradually with DS (Table 2). Overall, an increase in conductivity by about an order of magnitude from low DS (46%) to high DS (92%) was observed. The sample with 92% sulfonate groups exhibited the highest conductivity with secondary dopants (59.7 S cm^–1^) (Table 2, **Entry 5**), consistent with past reports for PEDOT:PSS with a PSS of a similar molecular weight and 100% sulfonation.^43^ We believe that the ability to finely control the degree of sulfonation using our method will allow for the precise adjustment of not only conductivity but also other crucial properties, such as wettability and adhesion. For example, partially sulfonated polystyrene has been shown to have improved wettability on silica surfaces.^43^ The potential to manipulate wettability and adhesion through this sulfonation method would provide benefits in the development of advanced materials for membrane technologies.^26,44^

**Table 2:**
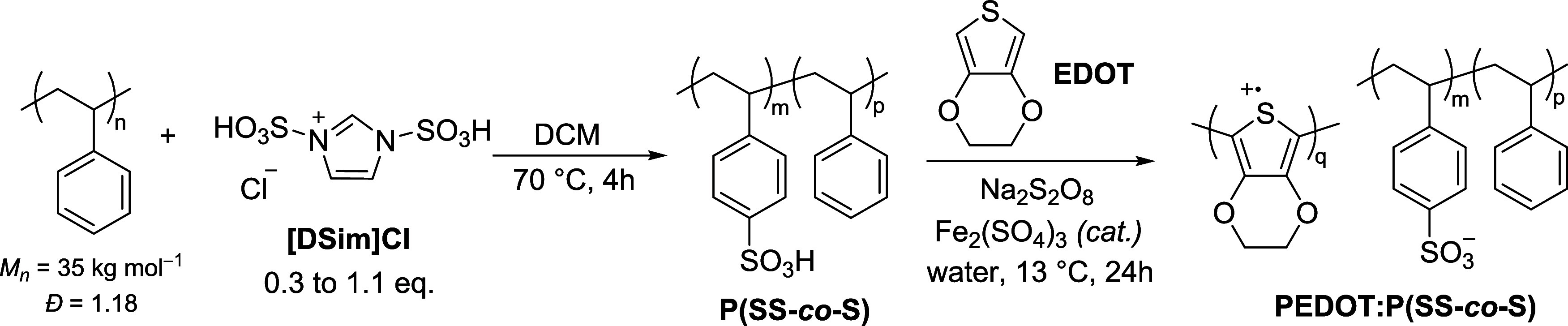
Control of DS of PS Using [Dsim]Cl and Conductivity of the Resulting PEDOT:P(SS-*co*-S) Polyelectrolyte Complexes

						conductivity (S cm^–1^)^c^	
entry	[Dsim]Cl/S molar ratio	DS^a^ (%)	isolated yield (%)	*M*_*n*_^b^ (kg mol^–1^)	*Đ^b^*	neat	5% DMSO, 0.1% DBSA
1	0.30	31	34	76.4	1.04	n.a.	n.a.
2	0.42	46	58	83.2	1.12	0.30 ± 0.04	6.4 ± 1.3
3	0.57	63	55	75.6	1.22	0.26 ± 0.02	25.3 ± 1.9
4	0.84	81	74	80.9	1.19	1.29 ± 0.03	34.5 ± 5.3
5	1.10	92	87	86.3	1.24	2.41 ± 0.16	59.7 ± 1.3

a Determined by UV–vis titration using 0.1 M NaOH.^35,36^

b Obtained by SEC in a water buffer:methanol 80:20 calibrated against PSSNa standards using a refractive index detector.

c Determined by four-point probe. Error corresponds to the standard deviation from the average of 3 independently prepared films from one PEDOT:PSS sample.

**Figure 4:**
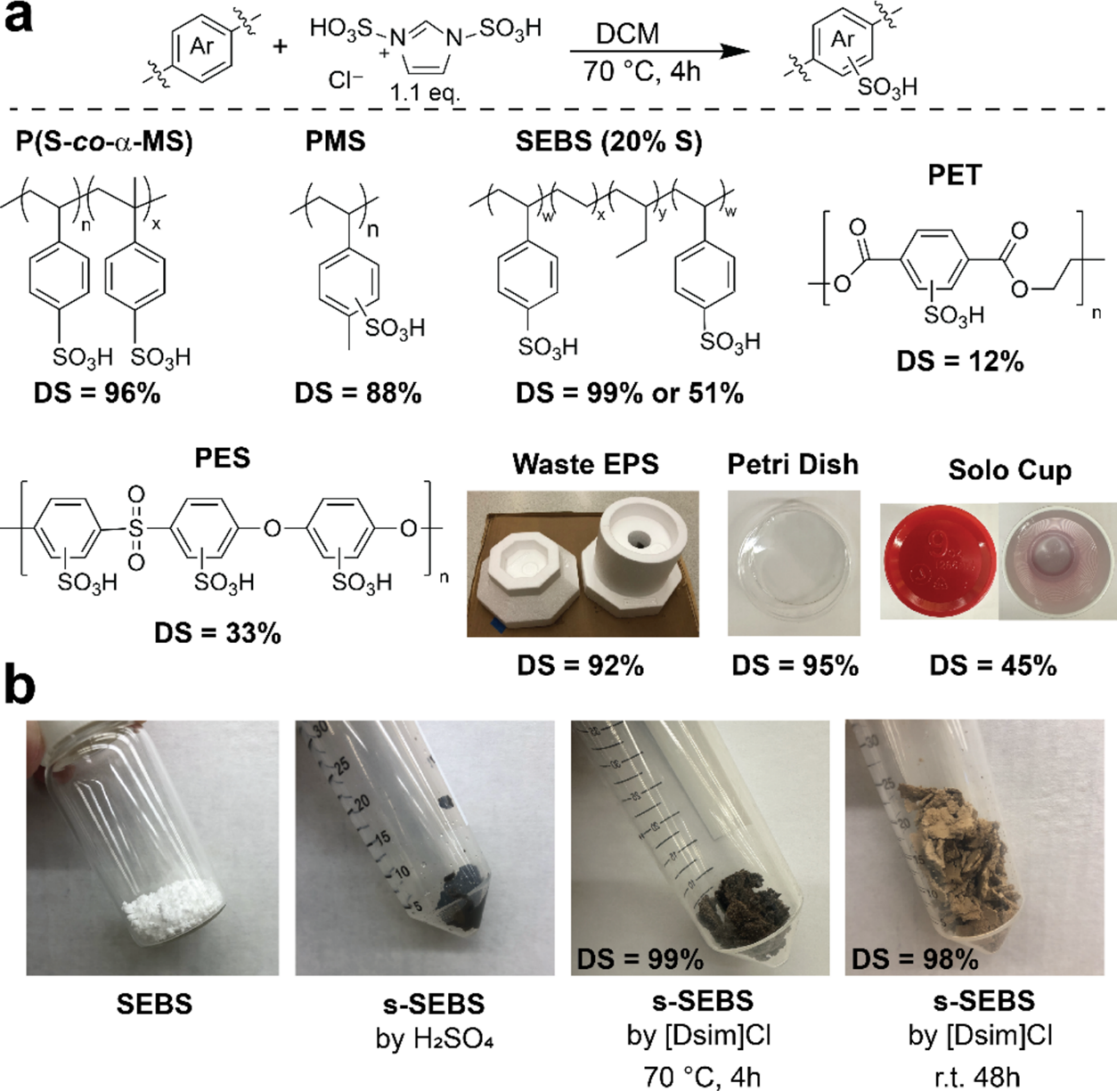
Imidazolium-mediated sulfonation of aromatic polymers. (a) Degree of sulfonation of commercially available aromatic polymers and waste PS plastic (EPS packaging, lab Petri dish, and solo cup) after reaction with [Dsim]Cl. (b) Photographs of styrene–ethylene–butylene–styrene (SEBS) before and after Vink’s sulfonation, and imidazolium-mediated sulfonation at 70 °C for 4 h or room temperature for 48 h.

After successfully sulfonating polystyrene using [Dsim]Cl, this approach was further applied to other common aromatic polymers using the optimized reaction conditions found for PS (Figure 4a). Owing to the moderate solubility of the resulting polymers in water, DS was determined using X-ray photoelectron spectroscopy (XPS) by calculating the C/S ratio (Figure S7). Sulfonation of the poly(styrene-*co*-α-methylstyrene) (P(S-*co*-α-MS)) copolymer and poly(4-methylstyrene) (PMS) afforded DS values of 96 and 88%, respectively. The thermoplastic elastomer styrene–ethylene–butylene–styrene (SEBS) led to a DS of 99%. This DS was higher than the previously reported sulfonation of SEBS using acetyl sulfate (<75%).^28^ We also compared our new approach to the Vink sulfonation method. We obtained DS > 100% for sulfonated-SEBS, indicating that it contained excess sulfuric acid which could not be removed by washing nor dialysis. As seen in Figure 4b, the physical characteristics of the resulting sulfonated-SEBS (s-SEBS) from [Dsim]Cl were also different from that of sulfuric acid. Using the imidazolium-mediated sulfonation resulted in a material that maintained its “rubbery” texture despite a dark brown coloration. This issue of coloration was partially addressed by sulfonating SEBS with [Dsim]Cl (Figure S8) at room temperature for 48 h. By conducting the reaction under milder conditions, the s-SEBS (DS = 98%, Figure S9) exhibited a light brown color and “rubbery” texture, rather than a sticky, black solid. The difference in physical aspect is potentially indicative of decreased side reactions occurring between the sulfonating agent and the plastic additives at room temperature. Furthermore, by adding 0.5 equiv of [Dsim]Cl, we successfully achieved a partial sulfonation of SEBS with DS = 51% (Figure S7d), highlighting that control over DS is not limited to PS. More challenging polymers such as polyether sulfone (PES) and poly(ethylene terephthalate) (PET) could also be sulfonated to 33 and 12% DS, respectively. For PES, DS was similar to that obtained using chlorosulfonic acid as a sulfonating agent (∼40%),^45^ but for PET, our approach led to a slightly lower DS compared with sulfonation using concentrated sulfuric acid (33%).^46^

Given these promising results with virgin PS resin and aromatic polymers, we then endeavored to demonstrate that the imidazolium-mediated sulfonation reaction was amenable to the upgrading of plastic waste to conducting polyelectrolyte complexes by sulfonating expanded polystyrene (EPS) obtained from waste packaging, a lab Petri dish, and a solo cup (Figure 4a). Using our standard conditions (70 °C for 4 h), we obtained PSS from EPS with a yield of 73% and DS of 94%. Similarly, using [Dsim]Cl at room temperature for 48 h (Table 3, **Entry 1**), PSS was successfully obtained with a 74% yield and a DS of 92% (Figures S10 and S11). The lower yield compared with commercial PS was likely due to the lower solubility of the resulting PSS in water, a result of the presence of cross-linking additives in EPS and high molecular weight polymer chains. We also successfully sulfonated a Petri dish to 95% using our standard conditions (70 °C for 4 h) with a good yield (Table 3, **Entry 2**); however, the resulting polymer was not soluble enough in water to get an SEC measurement, possibly due to its higher molecular weight compared with EPS. Lastly, we sulfonated a red solo cup at 70 °C for 4 h (Table 3, **Entry 3**). We obtained low DS (45% as measured by XPS, Figure S12), which can be attributed to the presence of dyes in the cup that were likely preferentially sulfonated. We believe the sulfonation of dyed plastic would be possible but would require further optimization or removal of the dye prior to sulfonation. PSS obtained from EPS waste was then used to prepare the conductive polyelectrolyte complex PEDOT:PSS, using the same conditions as above.^43^ As seen in Table 3, PEDOT:PSS obtained from the sulfonation of EPS exhibited a conductivity of 8.5 ± 0.4 S cm^–1^. This conductivity is slightly higher than that of commercially available PEDOT:PSS (undoped Clevios PH1000 is 0.1–1 S cm^–1^).^47^ As the composition (e.g., PSS molecular weight and dispersity) and specific synthetic conditions are unknown in commercial PEDOT:PSS, we cannot comment on the origin of this increase but it was on par with past experiments in our laboratory.^43^

**Table 3:** Sulfonation of Polystyrene Waste Using [Dsim]Cl and Electronic Properties of the PEDOT:PSS Resulting from Upgrading EPS

								conductivity (S cm^–1^)^e^		
entry	plastic waste precursor	PS *M*_*n*_^a^ (kg mol^–1^)	PS *Đ^a^*	yield (%)	DS (%)	PSS *M*_*n*_^d^ (kg mol^–1^)	PSS *Đ^d^*	neat	5% EG, 1% GOPS, 0.1% DBSA	C^*^ (F cm^–3^)^f^
1	EPS	38.5	3.5	74	92^b^	65.3	2.8	8.5 ± 0.4	74 ± 3.6	31.8 ± 0.7
2	petri dish	81.7	2.3	68	95^b^	N.S.^g^				
3	solo cup	64.8	3.0	55	45^c^	N.S.^g^				

a Obtained by SEC in THF calibrated against PS standards using a refractive index detector.

b Determined by UV–vis titration using 0.1 M NaOH.^35,36^

c Determined using X-ray photoelectron spectroscopy (XPS) by calculating the C/S ratio.

d Obtained by SEC in a water buffer:methanol 80:20 calibrated against PSSNa standards using a refractive index detector.

e Determined by four-point probe. Error corresponds to the standard deviation from the average of 6 independently prepared films from two independently synthesized PEDOT:PSS samples.

f Determined by cyclic voltammetry.^43^ Error corresponds to the standard deviation from the average of 3 independently prepared films from one PEDOT:PSS sample.

g N.S. signifies “not soluble” enough for SEC characterization.

**Figure 5:**
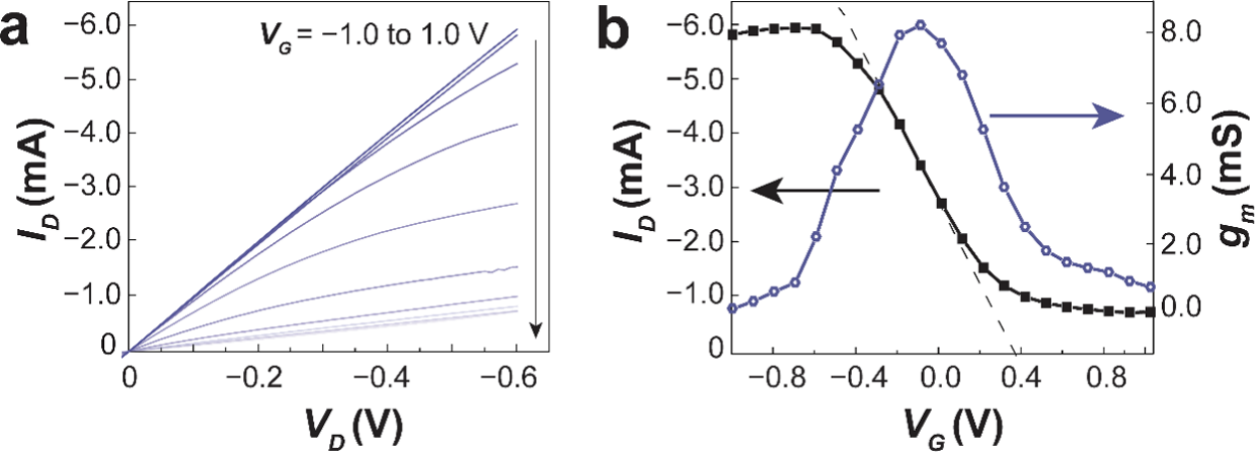
Performance of PEDOT:PSS prepared from sulfonated EPS in OECTs. (a) Output and (b) transfer curves.

To demonstrate that thisconducting polymer—comprised of roughly 2/3 upgraded plastic waste by mass—is efficient in electronic devices, we incorporated itin organic electrochemical transistors (OECTs) and silicon-based hybrid solar cells. For OECTs, the PEDOT:PSS samples were doped with 5 vol % ethylene glycol (EG), 1 vol % (3-glycidyloxypropyl)trimethoxysilane (GOPS), and 0.1 vol % DBSA, a combination of additives often used for OECT studies.^48−50^ We found that the electrical conductivity of PEDOT:PSS with these additives increased to 74 ± 3.6 S cm^–1^ (Table 3). Its volumetric capacitance (C*)—an important metric for the performance of OECTs—was measured by cyclic voltammetry (CV) at various film thicknesses (Figure S13).^43^ Upgraded PEDOT:PSS showed comparable C* to Clevios PH 1000 (31.8 ± 0.7 F cm^–3^, Table 3).^51^ The PEDOT:PSS sample obtained from EPS was used as a channel material for the organic electrochemical transistors (OECTs). Examples of output and transfer curves are shown in Figure 5a,b, respectively. The values of transconductance (*g*_m_), mobility-capacitance product ([μC*]), and charge transport mobility (μ_OECT_) measured and calculated from these device studies are tabulated in Table S2. Overall, PEDOT:PSS samples obtained from plastic waste sources performed similarly to Clevios PH 1000,^52,53^ with [μC*] of 113.6 ± 11.5 F cm^–1^ V^–1^ s^–1^ for EPS. These high values highlight the potential of using waste plastics as starting materials for the synthesis of high-performance organic mixed ionic-electronic conductors for OECTs.

Finally, we evaluated the efficiency of upgraded PEDOT:PSS as a hole transport layer (HTL) in silicon-based hybrid solar cells within the first degree of basic optimization. The device architecture (Figure S14) and fabrication of the photovoltaic cells are detailed in the Supporting Information (page S18). PEDOT:PSS obtained from EPS and Clevios PH1000 were used as HTL, and the results were compared. The devices were tested using illuminated current density–voltage measurements (*J*–*V*). The *J–V* performance of the fabricated devices was measured under 1.5 G standard illumination. The *J*–*V* response (Figure S15) was used to derive the most important cell parameters: open-circuit voltage (*V*_OC_), short-circuit current (*J*_SC_), fill factor (FF), and power conversion efficiency (PCE), for ease of comparison across the upgraded and commercial PEDOT:PSS (Table 4). PEDOT:PSS polymerized from the upgraded EPS was shown to perform at a level comparable to Clevios PH1000, albeit with a slightly lower PCE of around 6.9% compared with Clevios (8.4%). This lower PCE can be explained by a slight decrease in short-circuit current and FF. Nevertheless, these results show the potential of using waste plastics in hybrid photovoltaic devices.

**Table 4:** Silicon-Based Hybrid Solar Cell Parameters with a PEDOT:PSS HTL

HTL	*V*_OC_ (V)	*J*_SC_ (mA cm^–2^)	FF (%)	PCE (%)
PEDOT:PSS from EPS	0.56	23.9	51.5	6.9
Clevios PH1000	0.56	28	54.9	8.4

## Conclusions

An imidazolium salt, [Dsim]Cl, was successfully used as a sulfonating agent for a range of aromatic polymers, such as polystyrene. We demonstrated that the reaction can be performed at room temperature to achieve controlled degrees of sulfonation (DS) from 31 to 92%, which was not possible with previously reported sulfonation procedures. This control over the degree of sulfonation allowed for unique insights into structure–property relationships of partially sulfonated polymers, which was demonstrated here for conductive polymers but could also be impactful for studying wettability, adhesion, and membrane properties. This imidazolium-mediated procedure, while leading to high yields and DS, also proved milder, with lesser side reactions (e.g., cross-linking) and no decomposition of the main polymer chain. We showed that the majority of the imidazolium byproduct can be recovered and [Dsim]Cl regenerated to offset the potential economic and environmental disadvantage of using nitrogen-based reagents. A wide range of aromatic plastics were efficiently sulfonated, including expanded polystyrene, obtained from plastic waste packaging. We used this approach to upgrade plastic waste to a conducting polymer containing two-thirds of plastic waste by mass. This material proved efficient in organic electrochemical transistors and silicon-based hybrid solar cells, highlighting the potential of this sulfonation approach to access high-quality electronics from waste plastics. We expect that this approach will stimulate interest in developing sustainable approaches toward electronic materials through plastics-to-electronics upgrading.

## Supporting Information Available

Electronic supporting information (ESI) is available and includes experimental procedures for the synthesis of [Dsim]Cl; the sulfonation procedure; NMR of the monomers and polymers; SEC and XPS characterization of the polymers; comparison with past sulfonation methods; procedure for the recovery and regeneration of [Dsim]Cl; cyclic voltammetry results; OECT fabrication and results; and silicon-based hybrid solar cells fabrication and analysis (PDF)
