# Supplementary material for: Imidazolium-Based Sulfonating Agent to Control the Degree of Sulfonation of Aromatic Polymers and Enable Plastics-to-Electronics Upgrading
Source: JACS Au. 2024 Jul 3;4(7):2596–605. doi: 10.1021/jacsau.4c00355 (PMC11267550; doi:10.1021/jacsau.4c00355)
Supplement: Supplementary file 1 — au4c00355_si_001.pdf [file au4c00355_si_001.pdf]

Supplementary Information for

Imidazolium-based sulfonating agent to control the degree of sulfonation of aromatic polymers and enable plastics-to-electronics upgrading

Chun-Yuan Lo,[1] Kelsey P. Koutsoukos,[2] Dan My Nguyen,[1] Yuhang Wu,[2] David Alejandro Angel Trujillo,[2] Tabitha Miller,[3] Tulaja Shrestha,[1] Ethan Mackey,[1] Vidhika S. Damani,[2] Uddhav Kanbur,[3] Robert Opila,[2] David C. Martin,[2,4] David Kaphan,[3] and Laure V. Kayser*[1,2]

[1]Department of Chemistry and Biochemistry, University of Delaware, Newark, Delaware, 19716

[2]Department of Materials Science and Engineering, University of Delaware, Newark, Delaware, 19716

[3]Chemical Sciences and Engineering Division, Argonne National Laboratories, Lemont, Illinois, 60439

[4] Department of Biomedical Engineering, University of Delaware, Newark, Delaware, 19716

*Author to whom correspondence should be addressed: lkayser@udel.edu

GENERAL

Materials

Polystyrene (PS) (M_n = 35 kg mol^{-1}, $Đ$ = 1.18 and M_n = 120 kg mol^{-1} , $Đ$ = 1.13) were purchased from Polymer Source. Expanded polystyrene (EPS) was used directly from a packaging waste stream (Fisher 4L solvent package). Poly(4-methylstyrene) (M_w = 72 kg mol^{-1}), poly(styrene-co-α-4-methylstyrene), styrene-ethylene-butylene-styrene (SEBS) (M_w = 118 kg mol^{-1}), imidazole, chlorosulfonic acid, 98% sulfuric acid, styrene, n-butyllithium solution 1.6 M in hexanes, 3,4-ethylenedioxythiophene (EDOT), sodium persulfate ($Na_2S_2O_8$), iron(III) sulfate ($Fe_2(SO_4)_3$), dimethyl sulfoxide (DMSO), ethylene glycol (EG), dodecylbenzenesulfonic acid (DBSA), (3-glycidyloxypropyl) trimethoxysilane (GOPS), and acidic resin (Dowex Marathon C hydrogen form), were purchased from Sigma-Aldrich and used without further purification. Polyether sulfone (PES) (M_w = 6 kg mol^{-1}) and poly(ethylene terephthalate) (PET) (M_w = 100 kg mol^{-1}) were purchased from Polymer source, Inc. Surfactant Capstone FS-30 (non-ionic fluorosurfactant) was provided by Chemours. Distilled water filtered using a Milli-Q purification system was used throughout. Screen-printed electrodes (SPE) (catalog NO. C233BT) were purchased from Metrohm. These consist of a round gold working electrode at a diameter of 1.6 mm, surrounded

by a gold counter electrode and a silver pseudoreference electrode (-0.133 V vs. Ag/AgCl). The connection tracks are made of silver. Interdigitated electrodes (IDE) (catalog NO. G-IDEAU5) were also purchased from Metrohm, with two 5-µm-wide interdigitated gold electrodes with two gold connection tracks.

Size exclusion chromatography (SEC)

Number-average (M_n) and weight-average (M_w) molecular weights and dispersity of poly(styrene sulfonate) (PSS) were determined by HLC-8420 GPC EcoSEC LC system running in 20 % methanol and 80 % 0.3 M $NaNO_3$, 0.01 M NaH_2PO_4 in water at 40 °C (0.8 mL min^{-1}), using two PL aquagel-OH Mixed-H (8µm 50*7.5) columns, and calibrated against narrow polydispersity PSS standards (purchased from Polymer Standards Service).

Molecular weights and dispersity of PS were determined in reference to PS standards (purchased from Polymer Laboratories) using SEC with tetrahydrofuran (1.0 mL min^{-1}) as the eluent, using a HLC-8320 GPC EcoSEC equipped with TSKgel GMHhr-N column (5µm, 7.8 × 300 mm) in series with a RI-8320 refractive index (RI) detector.

Nuclear magnetic resonance (NMR)

^{1}H NMR spectra were collected on a Bruker 400 MHz spectrometer. The polymers were dissolved in D_2O or DMSO-d_5. The regioselectivity experiments were done by collecting solution phase ^{13}C NMR spectra using a Bruker UltraShield 500 MHz spectrometer (^{13}C NMR = 125 MHz). Chemical shifts for ^{13}C spectra were referenced using internal solvent resonances and are reported relative to tetramethylsilane (TMS). Quantitative ^{13}C NMR spectra were acquired with inverse gated decoupling; the zgig30 pulse sequence was employed with a delay of 8 s and an average of at least 15000 scans was used. Spectra were then processed using Mnova by Mestrelab Research. Phase correction followed by baseline correction (Bernstein polynomial) was performed prior to integration of the peaks.

METHODS

Synthesis of the sulfonating agent, 1,3-disulfonic acid imidazolium chloride ([Dsim]Cl)

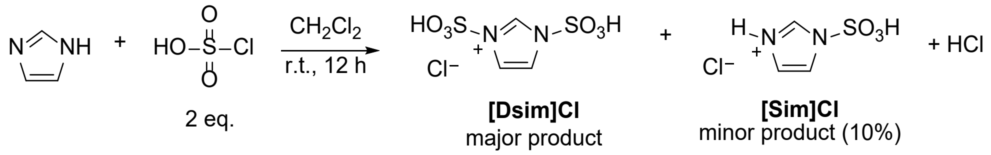

The sulfonating agent, [Dsim]Cl, was synthesized under strictly anhydrous conditions and characterized by adapting a previously reported procedure.[1,2] To a round-bottomed flask (100 mL) containing imidazole (1.02 g, 15 mmol) in dry dichloromethane (DCM) (100 mL), was added chlorosulfonic acid (2.1 mL, 3.6 g, 30.8 mmol, d = 1.75 g/mL) dropwise over a period of 20 min at room temperature. After the addition was completed, the reaction mixture was stirred for 12 h. The progress of the reaction was monitored by Fourier-transform infrared spectroscopy (FTIR). The residual oil was washed with anhydrous dichloromethane (3×50 mL) and dried under vacuum for overnight to give [Dsim]Cl as a viscous pale-yellow oil in 95% yield (3.55 g). The oil was characterized by Fourier transform infrared (FTIR) spectroscopy (**Figure S1a**) and proton nuclear magnetic resonance spectroscopy (^{1}H NMR, **Figure S1b**). The FTIR spectrum showed a characteristic broad peak at 3192 cm^{-1} indicating the presence of two OH groups of a SO_3H moiety in the ionic liquid. The bands at 1626 and 1588 cm^{-1} were assigned to C=C and C=N stretching vibrations, respectively, whereas the stretching band of the SO_3H group appeared at 1219 cm^{-1}. The other peaks at 1179, 1050, and 936 cm^{-1} were assigned to S−O symmetric stretching, S−O antisymmetric stretching, and N−S stretching vibrations, respectively, whereas the band centered at 576 cm^{-1} was attributed to the bending vibration of the SO_3H group. The ^{1}H NMR spectra (**Figure S1b**) is consistent with [Dsim]Cl, however it also showed 11% of the mono-sulfonated and protonated imidazolium, [Sim]Cl, as a side product. Our best efforts to limit this side product (e.g., by quenching excess HCl), were unsuccessful. We therefore proceeded with the sulfonation of aromatic polymers by accounting for this impurity, present in ~10% in each batch of [Dsim]Cl, when calculating the stoichiometric ratio of [Dsim]Cl/S.

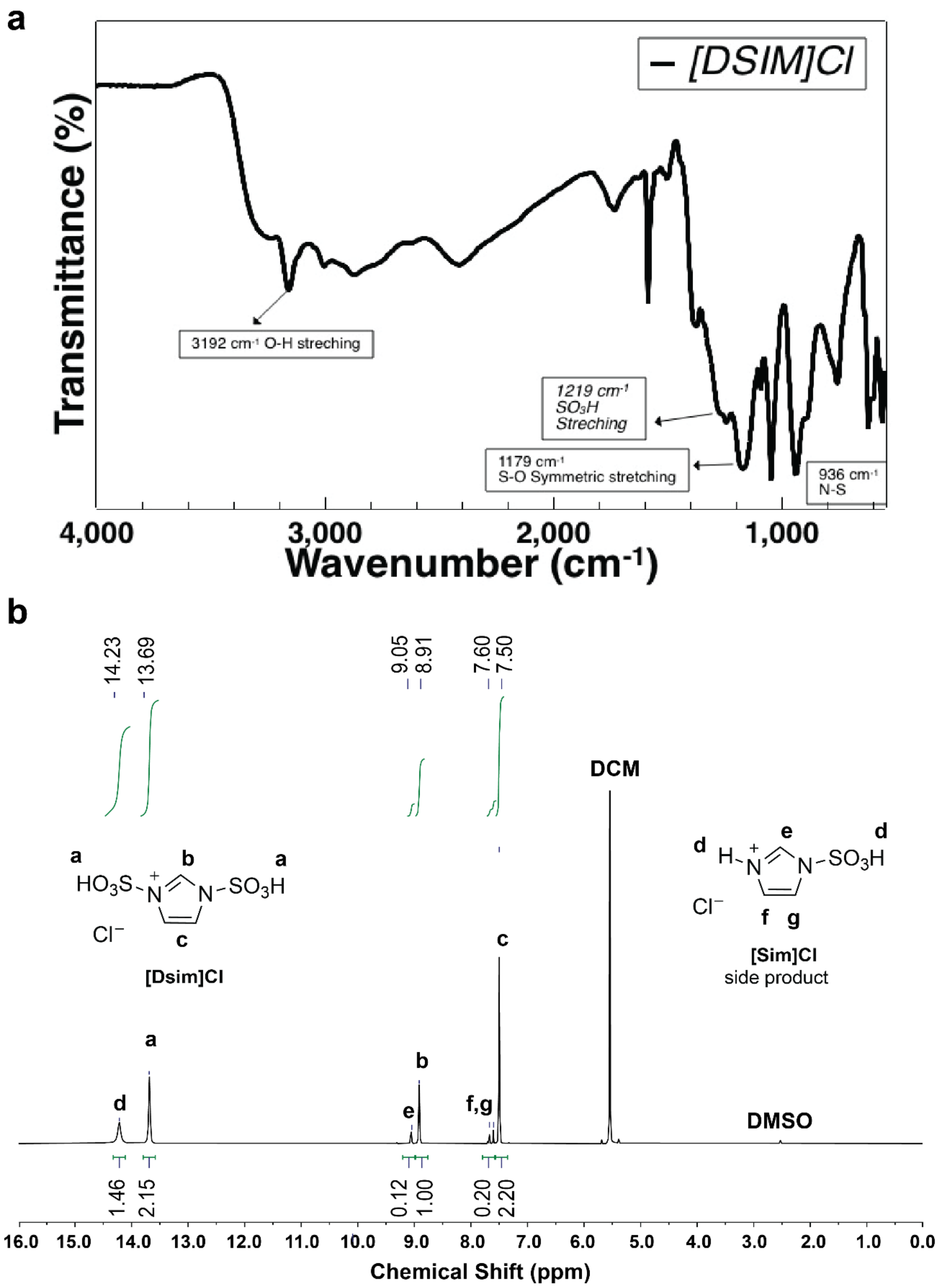

Figure S1. Characterization of [Dsim]Cl. **(a)** FTIR spectrum. **(b)** ^{1}H NMR spectrum showing 11% [Sim]Cl impurity.

General procedure for the sulfonation of PS by [Dsim]Cl.

Polystyrene (PS) (0.5 g, 4.8 mmol of styrene repeat units) was dissolved in 150 mL of dichloromethane (DCM). The sulfonating agent, [Dsim]Cl (90% purity, 1.4 g, n([Dsim]Cl) = 5.3 mmol), was added dropwise to the mixture at room temperature in a glovebox. The reaction was left to react for 4 h at 70 °C, during which time the sulfonated polymer precipitated. The reaction was stopped by adding 50 mL of DI water to dissolve the precipitate. The sulfonated polymer was extracted from the DCM layer with DI water (3×10 mL) using a separatory funnel. The combined water-soluble PSS fractions were purified by dialyzing for 2 days against DI water using a tubing with a molecular weight cut-off (MWCO) of 3500, then stirred over 100 mL of an acidic resin (Dowex Marathon C hydrogen form) for 60 min to remove excess imidazole. The polymers were then dried under vacuum.

General procedure for the sulfonation of PS by Vink's approach.

Polystyrene (PS) (1.0 g) was dissolved in 100 mL of cyclohexane, and incrementally added to a mixture containing 6 g of P_2O_5 and 28 mL of H_2SO_4 maintained at a temperature of 50 °C. This mixture was continuously stirred for four hours and then left undisturbed for an hour. The mixture was then cooled in an ice bath, and 17 g of ice-cold water were added. This procedure resulted in the formation of a yellowish-white, sticky substance. The resultant product was repeatedly rinsed with cooled distilled water, then dissolved in 300 mL of water, and finally was purified by dialyzing for 2 days against DI water using a tubing with a MWCO of 3500. The polymer was then dried under vacuum.

General procedure for the determination of the degree of sulfonation by titration.

We first used UV-Vis spectroscopy to accurately measure the concentration of the PSS solution for the titration. A calibration was used to determine the mass extinction coefficient of the materials (1.85 mL/mg at 262 nm) using the commercial PSS samples purchased from sigma.[3,4] Once the concentration was precisely determined, sulfonated polystyrene (0.1 to 0.2 g) and 3 drops of phenolphthalein solution as a pH indicator were added to a vial (20 mL) with DI water (5 mL). A 0.1 M solution of $NaOH_{(aq)}$ was then slowly added at room temperature. The color change of phenolphthalein indicated the end-point of the titration. The degree of sulfonation is defined as the mole percentage of sulfonated styrene units, which can be expressed by the following equation:[4]

$$Degree\ of\ sulfonation = \left[N * \frac{V}{\frac{W - 81 * N * V}{104}} \right] * 100.$$

where N (mol/L) and V (L) are the concentration and the titration volume of sodium hydroxide solution, respectively. W (g) is the weight of sulfonated polystyrene sample, 104 and 81 are the molar mass of the styrene unit and $–SO_3H$ groups respectively.

Table S1. Comparison between common sulfonation methods and this imidazolium-mediated sulfonation.

**Entry**	**Sulfonating agent, conditions**	**Isolated Yield**	**DS**	***Đ***	***para:meta ratio***	***Ref***
**1**	$SO_{3\,(l)}$ with P_2O_5, r.t.	Not reported	95%	--	--	*Turbak* [5] *Brown*[6]
**2**	Acetyl sulfate, 80 °C	42%<br>22%	40%<br>97%	--	--	*Martins*[7]
**3**	Chlorosulfonic acid, 50 °C	Not reported	62%	Not reported, but 10-70% degradation	--	*Akovali*[8]
**4**	96.7 wt% Sulfuric acid, 90 °C for PS <100 µm	98%	>95%	1.10	94:6	*Coughlin*[9]
**5**	Vink's approach[10] Sulfuric acid with P_2O_5, 40 °C	64%	91%	1.22	>98:2 [a]	*N.A.*
**6**	[Dsim]Cl, 70 °C	87%	92%	1.24	>99:1 [a]	*This work*

[a] Determined by quantitative ^{13}C NMR in D_2O (Figure S2).

Determination of the regioselectivity by quantitative ^{13}C NMR.

In the ^{13}C NMR of PSS (**Figure S2**), the expected peaks for poly(4-styrene sulfonate) (*para* sulfonation) were observed at 148, 140, 128, 125 ppm, and 40ppm.[9] According to past studies,[9] the peaks at 146, 142, 130, and 123 ppm correspond to carbon shifts resulting from sulfonation at the *meta* position. Comparison of the Vink and [Dsim]Cl sulfonation approaches show that both possessed excellent regioselectivity for the *para* position, 98.9 ± 0.2% and 99.5 ± 0.3%, respectively. The average Signal/Noise ratio was 0.09 and 0.52, respectively.

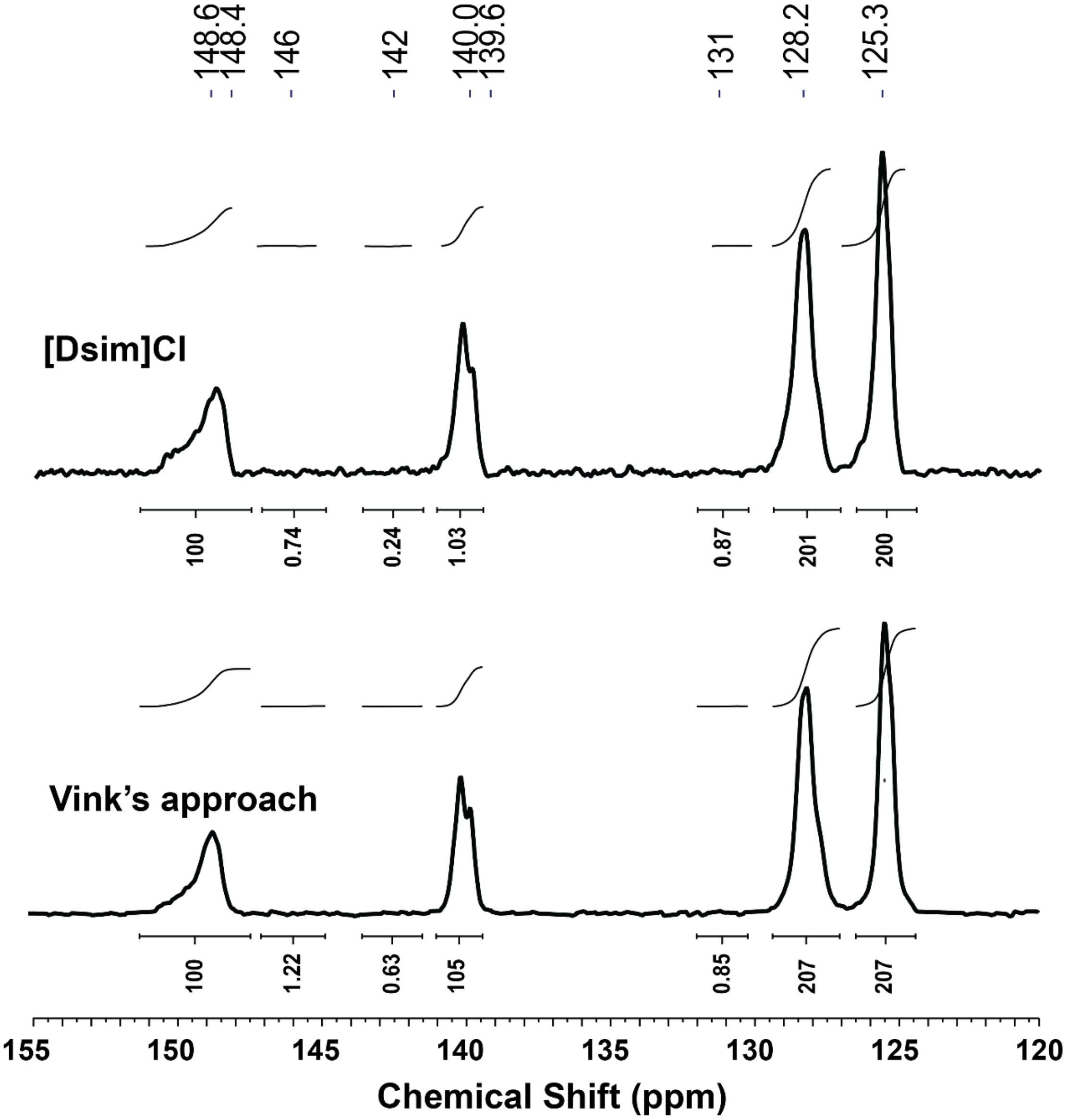

Figure S2. ^{13}C NMR spectra of PSS samples obtained by sulfonation of PS using [Dsim]Cl (top) or Vink sulfonation (bottom).

Recovery and regeneration of [Dsim]Cl.

To recover imidazole and regenerate [Dsim]Cl after the sulfonation of PS (0.5 g) with the [Dsim]Cl (90% purity, 1.45 g, n([Dsim]Cl) = 5.48 mmol), PSS was extracted from the reaction mixture in water and purified by dialysis (MWCO 3500) as described in the general procedure above. During the dialysis, the water-soluble small molecule by-product, identified as a protonated imidazolium salt (**Figure S3**), leached out of the dialysis tube. We note that the chemical shifts of the imidazolium protons shift with water content in the DMSO solvent as seen in the ^{1}H NMR below. This imidazolium was concentrated in vacuo then converted to imidazole by the addition of 50 mL 0.1M NaOH, and subsequently extracted in ethyl acetate (3 times with 50 mL). After evaporation of the solvent, pure imidazole (0.26 g, 3.8 mmol) (**Figure S4**) was obtained which can be re-used for the synthesis of [Dsim]Cl (90% yield but contained 17 mol% [Sim]Cl, **Figure S5**). Overall, we were able to recover and regenerate 66% of the [Dsim]Cl sulfonating agent.

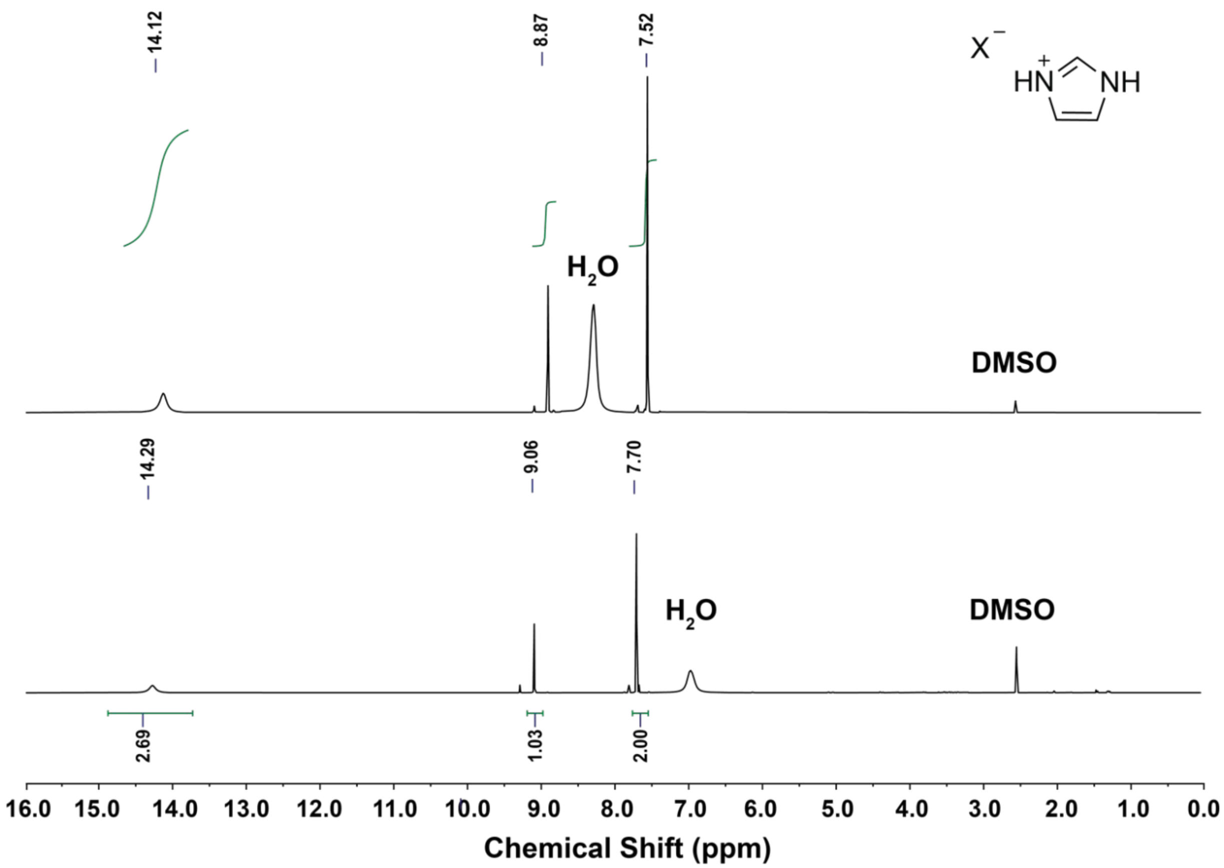

Figure S3. ^{1}H NMR of the protonated imidazolium by-product obtained after the reaction and recovered from outside the dialysis tube, containing high (top) or low (bottom) levels of water in DMSO-d_6.

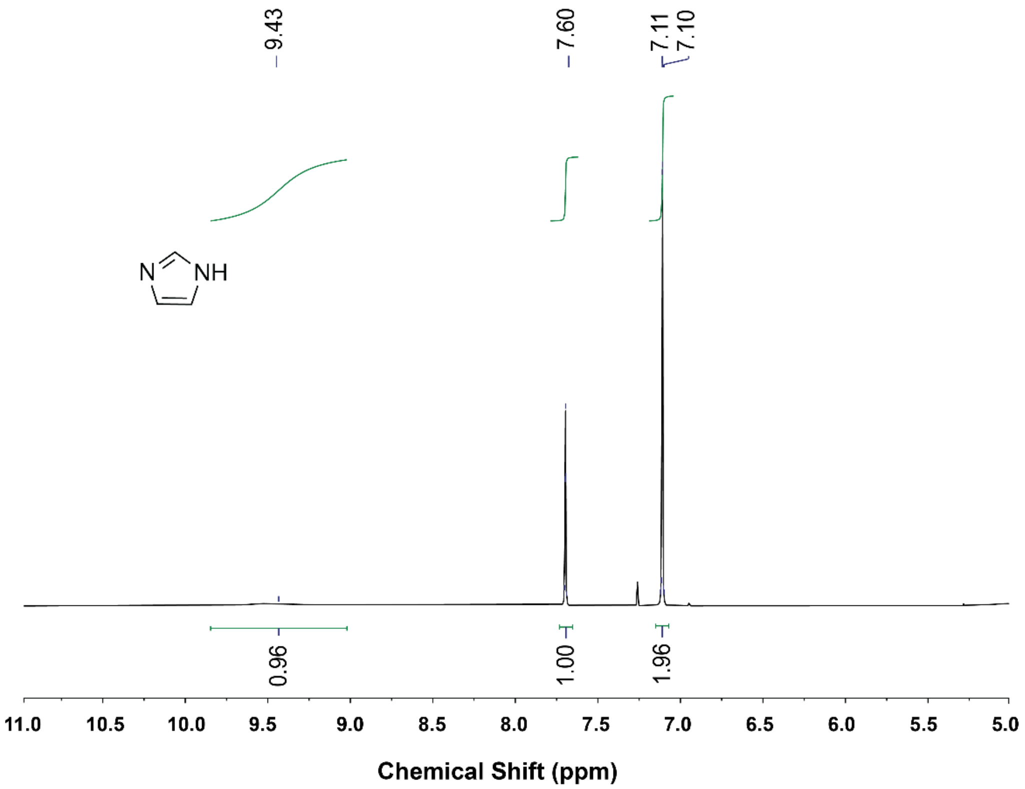

Figure S4. ^{1}H NMR of the recovered imidazole in $CDCl_3$.

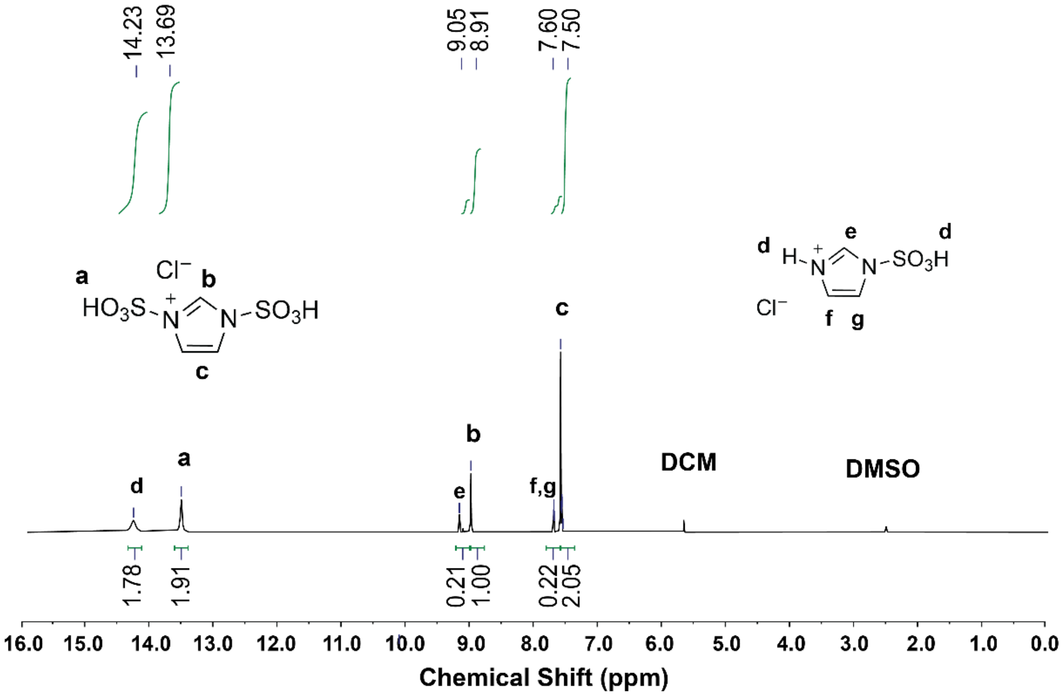

Figure S5. ^{1}H NMR of the regenerated [Dsim]Cl in DMSO-d_6, showing 17% [Sim]Cl side product.

^{1}H NMR of partially sulfonated PS to form [P(SS-co-S)].

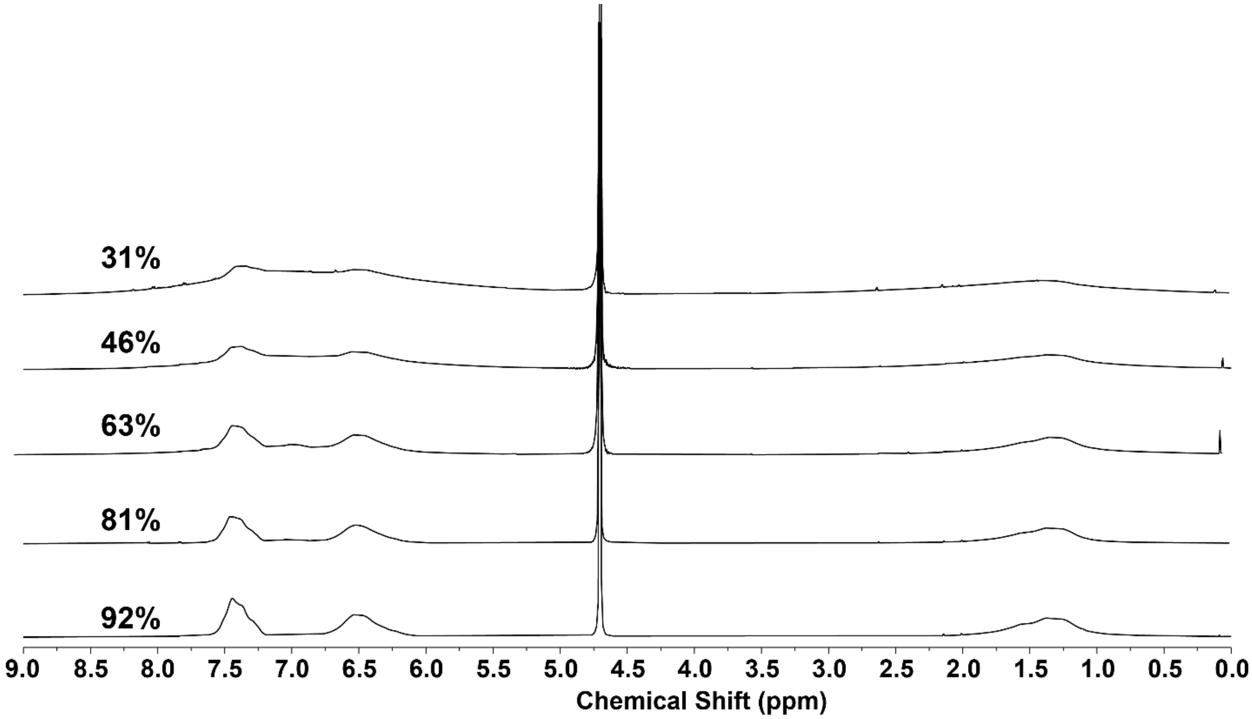

Figure S6. ^{1}H NMR of the partially sulfonated PS [P(SS-co-S)] with DS= 31, 46, 63, 81, and 92% in D_2O.

Synthesis of PEDOT:P(SS-*co*-S) and PEDOT:PSS from EPS.

P(SS-co-S) (0.14 g, 0.82 wt %) or PSS (0.14 g, 0.82 wt %) was dissolved in 13 g of DI water and stirred until fully dissolved. Then, EDOT (42 µL, 1.07 mmol) was added and stirred vigorously for 10 min at 13 °C before the addition of 2 mL of DI water with sodium persulfate (130 mg, 1.2 mmol) and 10 wt % iron (III) sulfate solution (30 µL, 0.2 mmol). The reaction was stirred at 13 °C for 24 hours. Then, PEDOT solution was purified over 3.6 mL of acidic (Dowex Marathon C hydrogen form) and 2 mL of basic (Lewatit MP-62 free base) resins for 1 h at room temperature and then filtered through a 10 µm filter.

General procedure for the sulfonation and characterization of aromatic polymers.

Aromatic polymers (0.5 g) were dissolved in 150 mL of DCM. The sulfonating agent, [Dsim]Cl (~1 eq. per aromatic repeat unit), was added dropwise to the mixture at room temperature and left to react for 4 h at 70 °C. The reaction was stopped by adding 50 mL of DI water and was filtered via a 10 µm PTE filter to collect the solids. The purification process for water-soluble products followed the same procedure as described above for the sulfonation of PS. Water-insoluble sulfonated products were purified by dialysis against deionized water over a period of two days, using a tubular membrane with a MWCO of 3500. Subsequently, the solid polymer was stirred in 0.1 M NaOH solution followed by 0.1 M HCl solution for 30 minutes each, for multiple iterations, and then thoroughly rinsed with DI water several times. Finally, the solid was dried under high vacuum for 24 h. The degree of sulfonation was determined by quantitative X-ray photoelectron spectroscopy from the Sulfur/Carbon atomic ratio (**Figure S7**).[11] The N1s region was also monitored to ensure that no residual imidazole by-products remained in the final polymers.

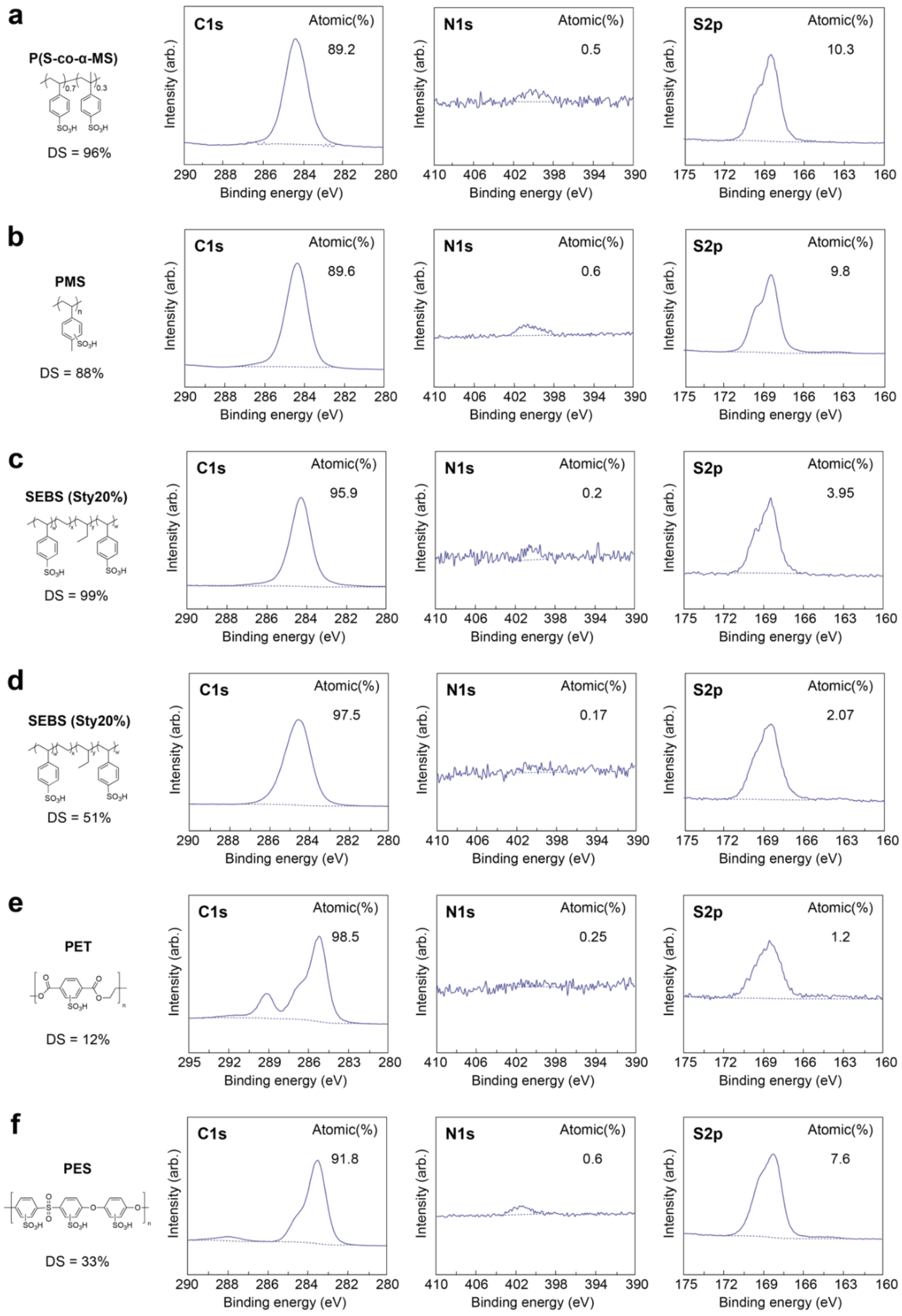

Figure S7. X-ray photoelectron spectroscopy of aromatic polymers after sulfonating with [Dsim]Cl focused on the C(1s), N(1s), and S(2p) regions. **(a)** P(S-co-α-MS), **(b)** PMS, **(c)** SEBS with 1 eq. [Dsim]Cl, **(d)** SEBS with 0.5 eq. [Dsim]Cl, **(e)** PET, and **(f)** PES.

Procedure for the sulfonation of SEBS at room temperature.

Styrene-ethylene-butylene-styrene (SEBS) (1.58 g, 20% of styrene, total includes 0.5 g of styrene) was dissolved in 150 mL of DCM. The sulfonating agent, [Dsim]Cl (69%, purity, 1.45 g, n([Dsim]Cl) = 5.5 mmol, **Figure S8**), was added dropwise to the mixture at room temperature and left to react for 48 h at room temperature. The reaction was stopped by adding 50 mL of DI water and was filtered via a 10 μm PTE filter to collect the solids. The purification process and characterization were identical to the ones above for insoluble aromatic polymers. The XPS of s-SEBS showed a DS of 98% (**Figure S9**).

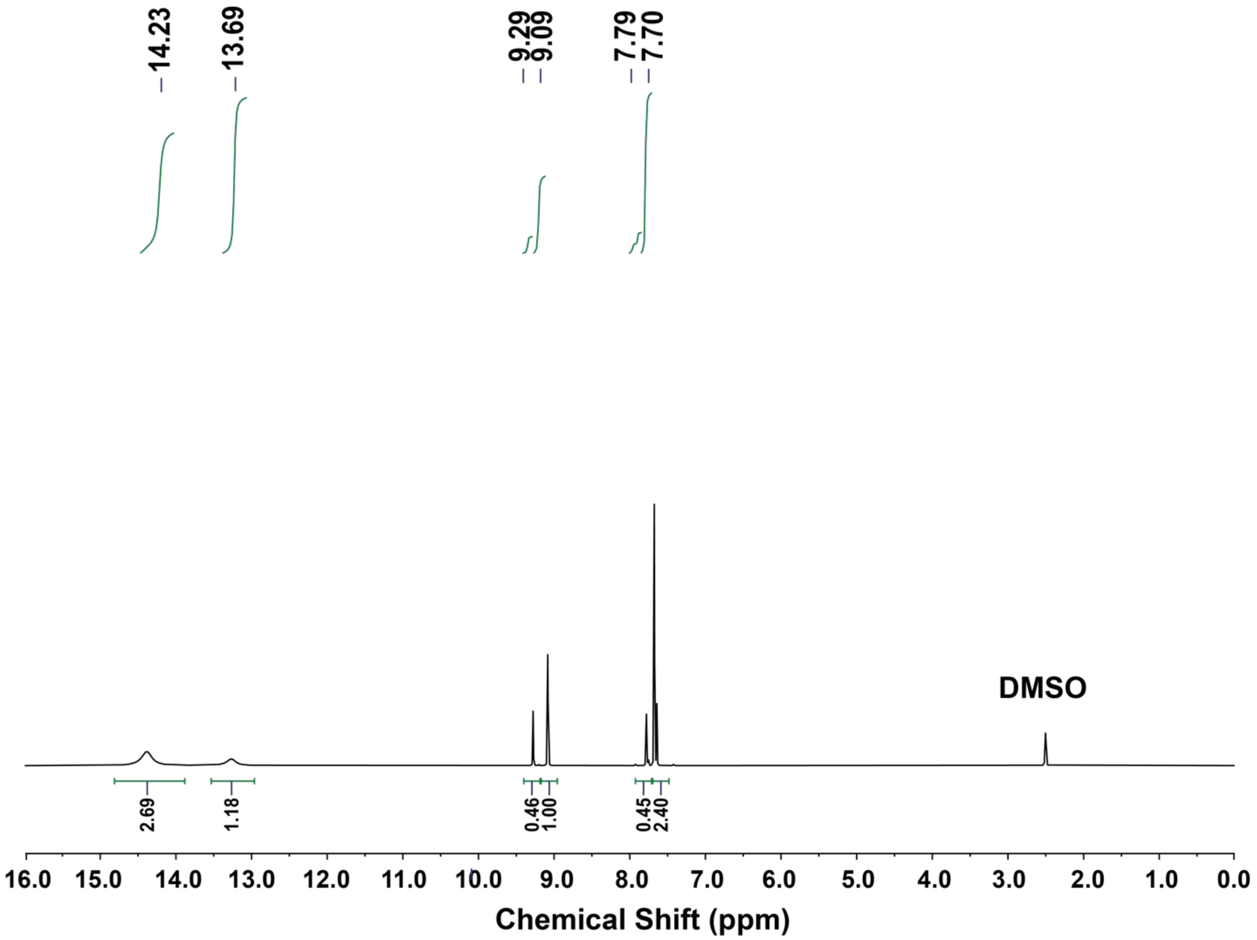

Figure S8. ^{1}H NMR spectrum in DMSO-d_6 of the sulfonating agent used in the room temperature sulfonation of SEBS and EPS.

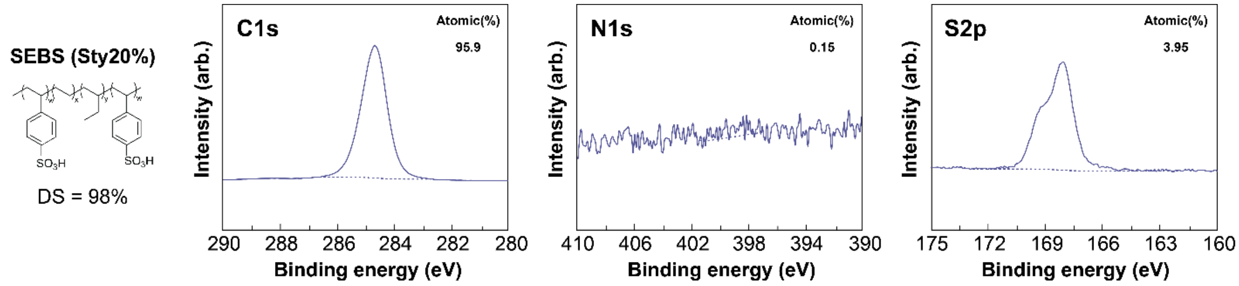

Figure S9. X-ray photoelectron spectroscopy focused on the C(1s), N(1s), and S(2p) regions of s-SEBS obtained from sulfonation at room temperature for 48h.

Procedure for the sulfonation of EPS at room temperature.

Expanded polystyrene (EPS) (0.5 g, 4.8 mmol of styrene repeat units) was dissolved in 150 mL of DCM. The sulfonating agent, [Dsim]Cl (69% purity, 1.45 g, n([Dsim]Cl) = 5.5 mmol, **Figure S8**), was added dropwise to the mixture at room temperature. The reaction was left to react for 48 h at room temperature, during which time, the sulfonated polymer precipitated. Then, 50 mL of DI water was added to dissolve the precipitate. The sulfonated polymer was then separated from the dichloromethane (DCM) layer by extracting it into DI water (three times with 10 mL each) using a separatory funnel. The combined aqueous phases containing the PSS were then purified through dialysis for two days against DI water, employing tubing with a MWCO of 3500. Subsequently, the solution was mixed with 100 mL of an acidic resin (Dowex Marathon C in hydrogen form) for 60 minutes. The polymer was dried under a vacuum to afford a DS of 92%.

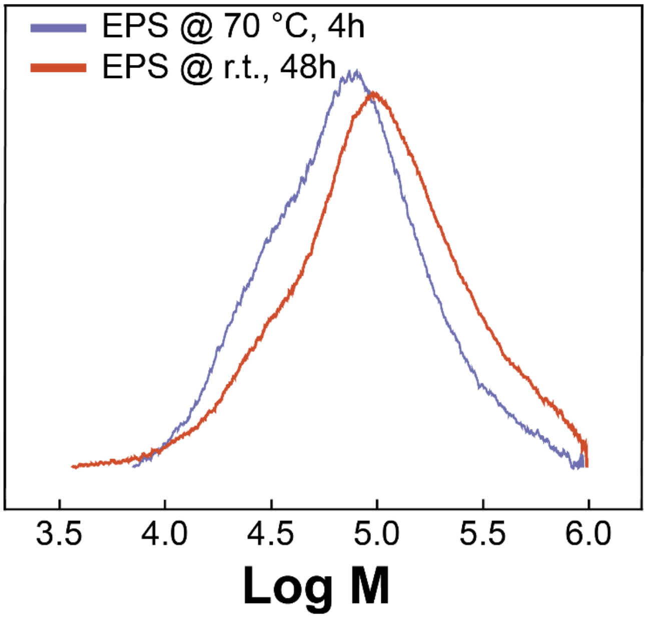

Figure S10. SEC selected PSS samples obtained by sulfonation of EPS with [Dsim]Cl at 70 °C for 4 h or room temperature for 48 h.

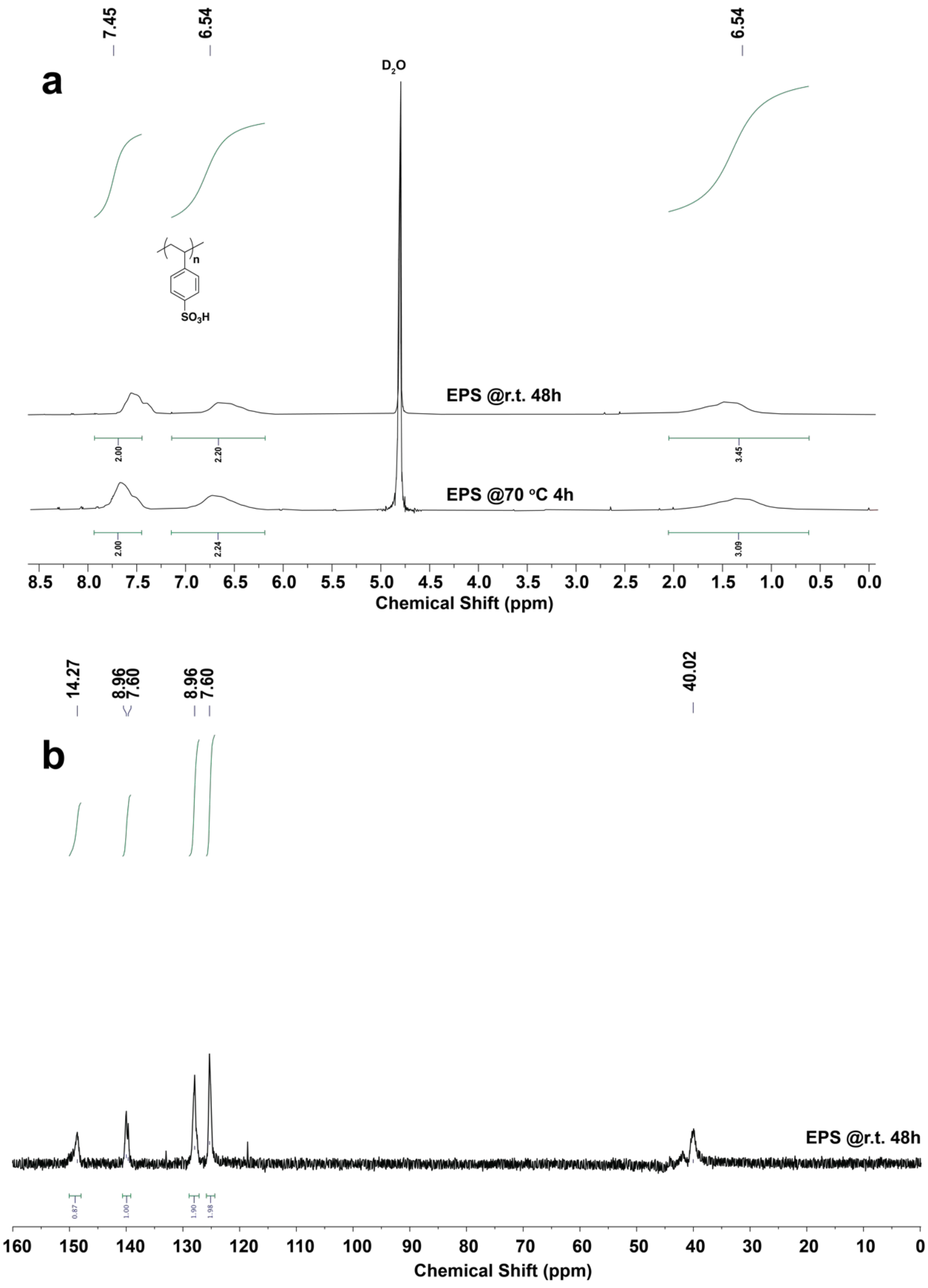

Figure S11. Sulfonation of EPS by [Dsim]Cl. **(a)** ^{1}H NMR spectra of PSS samples obtained by sulfonation of EPS at room temperature for 48 h (top) and 70 °C for 4 h (bottom). **(b)** ^{13}C NMR spectrum in D_2O.

XPS analysis of the sulfonated solo cup

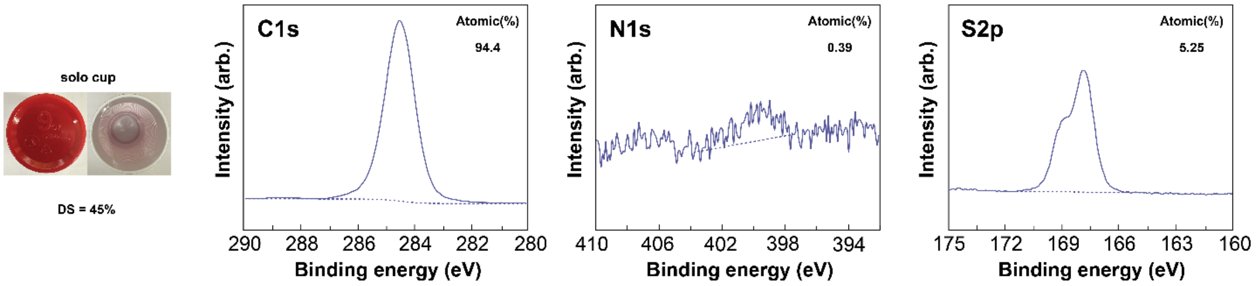

Figure S12. XPS analysis focused on the C(1s), N(1s), and S(2p) regions of PSS obtained from sulfonation at 70 °C of a solo cup.

Electronic performance of PEDOT:PSS from upgraded EPS.

To determine the volumetric capacitance (C^*), we performed cyclic voltammetry (CV) on the PEDOT:PSS samples at various film thicknesses (**Figure S13**). The OECTs were fabricated similarly to our previously reported work on screen printed electrodes.[12]

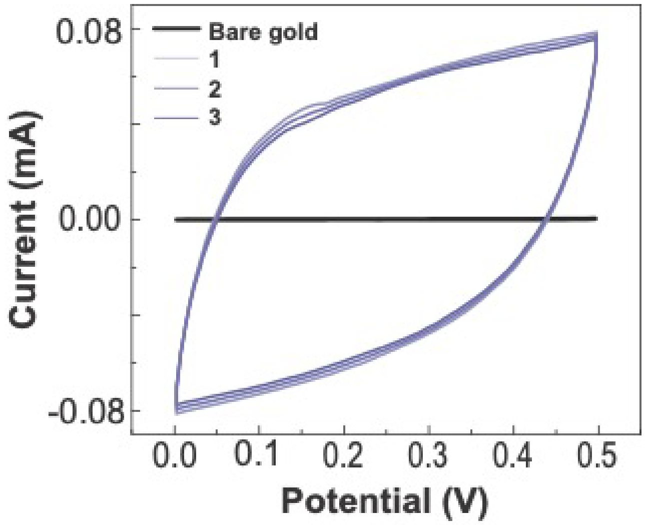

Figure S13. CV scans of PEDOT:PSS samples, with PSS obtained from EPS, with 5 vol% EG, 1 vol% GOPS, and 0.1 vol% DBSA with 1-3 spin-coated layers on IDE.

Table S2. Dimensions and figures of merit for the OECT devices.

Sample Name	WdL_c^{-1} (nm)	g_m (mS)	$(V_{Th} - V_G)$ (V)	$[\mu_{OECT}C^*]$ (F cm^{-1} V^{-1} s^{-1})	μ_{OECT} [a] (cm^2 V^{-1} s^{-1})
PEDOT:PSS from EPS	1113	7.5	0.65	113.6 ± 11.5	3.24
	1014	7.3	0.57		3.83
	1094	7.4	0.61		3.36

[a] Calculated by dividing $[\mu_{OECT}C^*]$ by the average C^* obtained by CV.

Hybrid photovoltaic devices fabrication and characterization.[13]

N-type silicon (100)-textured substrates (doped with phosphorous, CZ) with back surface field (BSF) were fabricated at Arizona State University (Solar Power Lab, ASU, Tempe, AZ, USA). These wafers were 145 μm thick with 1–5 Ω-cm resistivity. Random texturization was performed on both sides using potassium hydroxide (2% KOH yielding pyramid sizes of about 3–5 μm base size) alkaline etching. To create BSF n + layer on the n-type wafers, phosphorous oxychloride diffusion was performed at 820 °C, 15 min with a $POCl_3$ carrier gas flow rate of 1500 sccm (standard cubic centimeters per minute) for phosphosilicate glass (PSG) growth and dopant drive-in. Finally, a 10-min buffered oxide etch was used to remove the PSG. The sheet resistance value of the BSF side was 55 Ω/square.

In these studies, we used 7 wt% ethylene glycol (EG) as the co-solvent and 0.25 wt% Capstone FS-30 as the surfactant added to PEDOT:PSS. All wafers were cleaned using a Piranha etch (H_2SO_4:H_2O_2 = 4:1) for five minutes, followed by a 5-min DI water rise and a two-minute immersion in hydrofluoric acid (HF, 2 wt%). After cleaning, the substrates were blow-dried with nitrogen. The PEDOT:PSS dispersions with EG and Capstone FS-30 were then spin-coated on the front of the wafers (2250 rpm for 300 s) on a Headway Research spin coater, then immediately baked on a hot plate at 135 °C for 15 min. For complete hybrid solar cell devices (**Figure S14**), metal contacts were deposited using electron beam physical vapor deposition on a dual electron-beam evaporator (Wilmington, MA, USA). Aluminum (2μm) was used as the back contact and deposited on the BSF-treated side of the wafer, while silver (500nm) was used as the front contact and was deposited with the help of a finger patterned shadow mask directly on the PEDOT:PSS films.

The devices were tested using illuminated current density–voltage measurements (J–V) (**Figure S15**). JV response was measured by means of a DC source meter (Keithley 2400 sourcemeter, USA) both in the dark and light, under air mass 1.5G standard illumination.

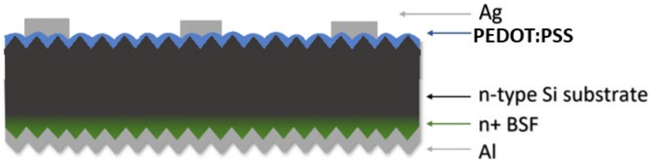

Figure S14. Schematic of hybrid silicon-based photovoltaic (PV) device architecture.

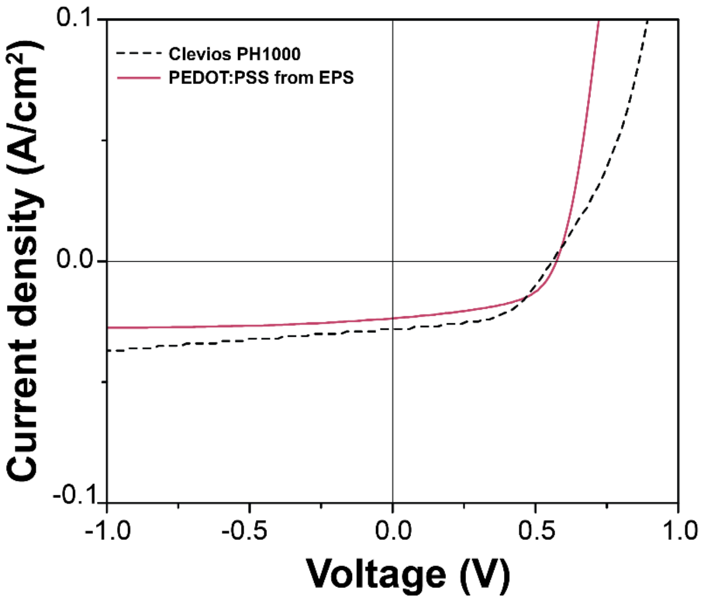

Figure S15. J–V performance of hybrid PV devices with a PEDOT:PSS HTL.

REFERENCES

1 Zolfigol, M. A., A. Khazaei, A. R. Moosavi-Zare, A. Zare, V. Khakyzadeh. Rapid Synthesis of 1-Amidoalkyl-2-Naphthols over Sulfonic Acid Functionalized Imidazolium Salts. *Appl. Catal. A Gen.*, **2011**, *400*, 70.

2 Zolfigol, M. A., V. Khakyzadeh, A. R. Moosavi-Zare, G. Chehardoli, F. Derakhshan-Panah, A. Zare, O. Khaledian. Novel Ionic Liquid 1,3-Disulfonic Acid Imidazolium Hydrogen Sulfate {[Dsim] HSO4 } Efficiently Catalyzed N-Boc Protection of Amines.

Sci. Iran., **2012**, *19*, 1584.

3 Sen, A. K., S. Roy, V. A. Juvekar. Effect of Structure on Solution and Interfacial Properties of Sodium Polystyrene Sulfonate (NaPSS). *Polym. Int.*, **2007**, *56*, 167.

4 Bekri-Abbes, I., S. Bayoudh, M. Baklouti. Converting Waste Polystyrene into Adsorbent: Potential Use in the Removal of Lead and Cadmium Ions from Aqueous Solution. *J. Polym. Environ.*, **2006**, *14*, 249.

5 Turbak, A. F. Polymer Sulfonation without Cross Linking. The Sulfur Trioxide-Phosphate System. *I&EC Prod. Res. Dev.*, **1962**, *1*, 275.

6 Brown, D. W., R. E. Lowry. Molecular Weight Standards from Sulfonation of Polystyrene. *J. Polym. Sci. Polym. Chem. Ed.*, **1979**, *17*, 1039.

7 Martins, C. R., G. Ruggeri, M.-A. De Paoli. Synthesis in Pilot Plant Scale and Physical Properties of Sulfonated Polystyrene. *J. Braz. Chem. Soc.*, **2003**, *14*, 797.

8 Akovali, G., A. Özkan. Notes on Modification of Polystyrene by Sulphonation: Some Properties of Poly(Styrenesulphonic Acid). *Polymer*, **1986**, *27*, 1277.

9 Coughlin, J. E., A. Reisch, M. Z. Markarian, J. B. Schlenoff. Sulfonation of Polystyrene: Toward the "Ideal" Polyelectrolyte. *J. Polym. Sci. Part A Polym. Chem.*, **2013**, *51*, 2416.

10 Vink, H. A New Convenient Method for the Synthesis of Poly(Styrenesulfonic Acid). *Die Makromol. Chemie*, **1981**, *182*, 279.

11 Lufrano, F., G. Squadrito, A. Patti, E. Passalacqua. Sulfonated Polysulfone as Promising Membranes for Polymer Electrolyte Fuel Cells. *J. Appl. Polym. Sci.*, **2000**, *77*, 1250.

12 Lo, C. Y., Y. Wu, E. Awuyah, D. Meli, D. M. Nguyen, R. Wu, B. Xu, J. Strzalka, J. Rivnay, D. C. Martin, L. V. Kayser. Influence of the Molecular Weight and Size Distribution of PSS on Mixed Ionic-Electronic Transport in PEDOT:PSS. *Polym. Chem.*, **2022**, *13*, 2764.

13 Iyer, A., J. Hack, D. A. Angel Trujillo, B. Tew, J. Zide, R. Opila. Effects of Co-Solvents on the Performance of PEDOT:PSS Films and Hybrid Photovoltaic Devices. *Applied Sciences*. **2018**, *8*(11), 2052.
